# Spectroscopic characterization and assessment of microbiological potential of 1,3,4-thiadiazole derivative showing ESIPT dual fluorescence enhanced by aggregation effects

**DOI:** 10.1038/s41598-022-26690-1

**Published:** 2022-12-22

**Authors:** Iwona Budziak-Wieczorek, Lidia Ślusarczyk, Beata Myśliwa-Kurdziel, Martyna Kurdziel, Monika Srebro-Hooper, Izabela Korona-Glowniak, Mariusz Gagoś, Grzegorz Gładyszewski, Andrzej Stepulak, Dariusz Kluczyk, Arkadiusz Matwijczuk

**Affiliations:** 1 Department of Chemistry, Faculty of Food Science and Biotechnology, University of Life Sciences in Lublin, Akademicka 15, 20-950 Lublin, Poland; 2 Department of Biophysics, Faculty of Environmental Biology, University of Life Sciences in Lublin, Akademicka 13, 20-950 Lublin, Poland; 3 Department of Plant Physiology and Biochemistry, Faculty of Biochemistry, Biophysics and Biotechnology, Jagiellonian University, Gronostajowa 7, 30-387 Kraków, Poland; 4 Department of Theoretical Chemistry, Faculty of Chemistry, Jagiellonian University, Gronostajowa 2, 30-387 Kraków, Poland; 5 Department of Pharmaceutical Microbiology, Medical University of Lublin, 20-093 Lublin, Poland; 6 Department of Cell Biology, Maria Curie-Sklodowska University, Akademicka 19, 20-033 Lublin, Poland; 7 Department of Biochemistry and Molecular Biology, Medical University of Lublin, 20-093 Lublin, Poland; 8 Department of Applied Physics, Lublin University of Technology, Nadbystrzycka 38, 20-618 Lublin, Poland; 9 Department of Plant Physiology and Biophysics, Institute of Biological Sciences, Maria Curie-Sklodowska University, 20-033 Lublin, Poland

**Keywords:** Biologics, Medicinal chemistry, Biophysics, Drug discovery, Microbiology, Chemistry, Physics

## Abstract

In the presented study, advanced experimental techniques, including electronic absorption and fluorescence spectroscopies [with Resonance Light Scattering (RLS)], measurements of fluorescence lifetimes in the frequency domain, calculations of dipole moment fluctuations, quantum yields, and radiative and non-radiative transfer constants, were used to characterize a selected analogue from the group of 1,3,4*-*thiadiazole, namely: *4-[5-(naphthalen-1-ylmethyl)-1,3,4-thiadiazol-2-yl]benzene-1,3-diol* (NTBD), intrinsically capable to demonstrate *enol → keto* excited-states intramolecular proton transfer (ESIPT) effects. The results of spectroscopic analyses conducted in solvent media as well as selected mixtures were complemented by considering biological properties of the derivative in question, particularly in terms of its potential microbiological activity. The compound demonstrated a dual fluorescence effect in non-polar solvents, e.g. chloroform and DMSO/H_2_O mixtures, while in polar solvents only a single emission maximum was detected. In the studied systems, ESIPT effects were indeed observed, as was the associated phenomenon of dual fluorescence, and, as demonstrated for the DMSO: H_2_O mixtures, the same could be relatively easily induced by aggregation effects related to aggregation-induced emission (AIE). Subsequently conducted quantum-chemical (TD-)DFT calculations supported further possibility of ESIPT effects. The following article provides a comprehensive description of the spectroscopic and biological properties of the analyzed 1,3,4*-*thiadiazole derivatives, highlighting its potential applicability as a very good fluorescence probes as well as a compound capable of high microbiological activity.

## Introduction

One of the main objectives of contemporary medicine is to respond to the rapidly increasing incidence of cancer, neurodegenerative diseases, and a variety of maladies caused by bacteria and fungi^1–3^. Concern for fungal pathogens, which can cause serious invasive infections, is a limitation of treatment options to only three major classes of antifungal drugs. Over the last 2 decades no new classes of antifungal drugs have become available, and only one single new agent from a known antifungal class has been approved in the last decade. Recently, searching for new agents with antifungal activity has become more important because of the increase in frequency of severe fungal infections and the expansion of fungal resistance to drugs^4^. Selected derivatives from the group of *1,3,4-*thiadiazoles prove potentially beneficial effects in the treatment and prevention of said diseases^5,6^, and some of them have already found clinical applications^7,8^. Their unusual spectroscopic and crystallographic properties dictate a large spectrum of their additional applications; they can be used as laser pigments^9^, ligands complexing rare earth metal ions^10^, molecular probes^11^, as well as UV stabilizers^12^. Hence, detailed identification of the mechanisms responsible for the observed spectroscopic phenomena should significantly contribute to relating structural features with respective biological properties of the analogues in question.

The 1,3,4-thiadiazoles analyzed by our own as well as other research teams worldwide are characterized by a range of interesting spectroscopic, crystallographic, and biological properties^13^ that are also frequently cited as reasons for unusual pharmacological traits of the group’s analogues^14^. These features, often evidenced in spectroscopic measurements, include the ability to form complexes with rare earth metal ions^14^, aggregation effects^15^, four-stage process of phototautomerization, i.e. excited-state intramolecular proton transfer (ESIPT) process^16^. And phenomenon of dual fluorescence produced by a single excitation assigned usually in literature to the aforementioned ESIPT, possibility of intramolecular CT states, often accompanied by a partial twisting of the molecule (twisted intramolecular charge transfer (TICT))^17^, formation of excimer systems^18^, aggregation-induced emission (AIE)^19–21^, or emission related to inconsistency with the Kasha’s rule, as described by Brancato et. al.^22^. Among particularly interesting biological and pharmaceutical properties of the 1,3,4-thiadiazole derivatives studied by our team for a long time, one must mention the synergistic enhancement of their antimycotic properties when combined with antibiotics from the polyene antimycotic group, e.g. amphotericin B (AmB)^13^. Compositions showing the effects of synergistic activity are characterized by particularly high antimycotic effectiveness while at the same time reducing the AmB toxicity^13^.

**Figure 1:**
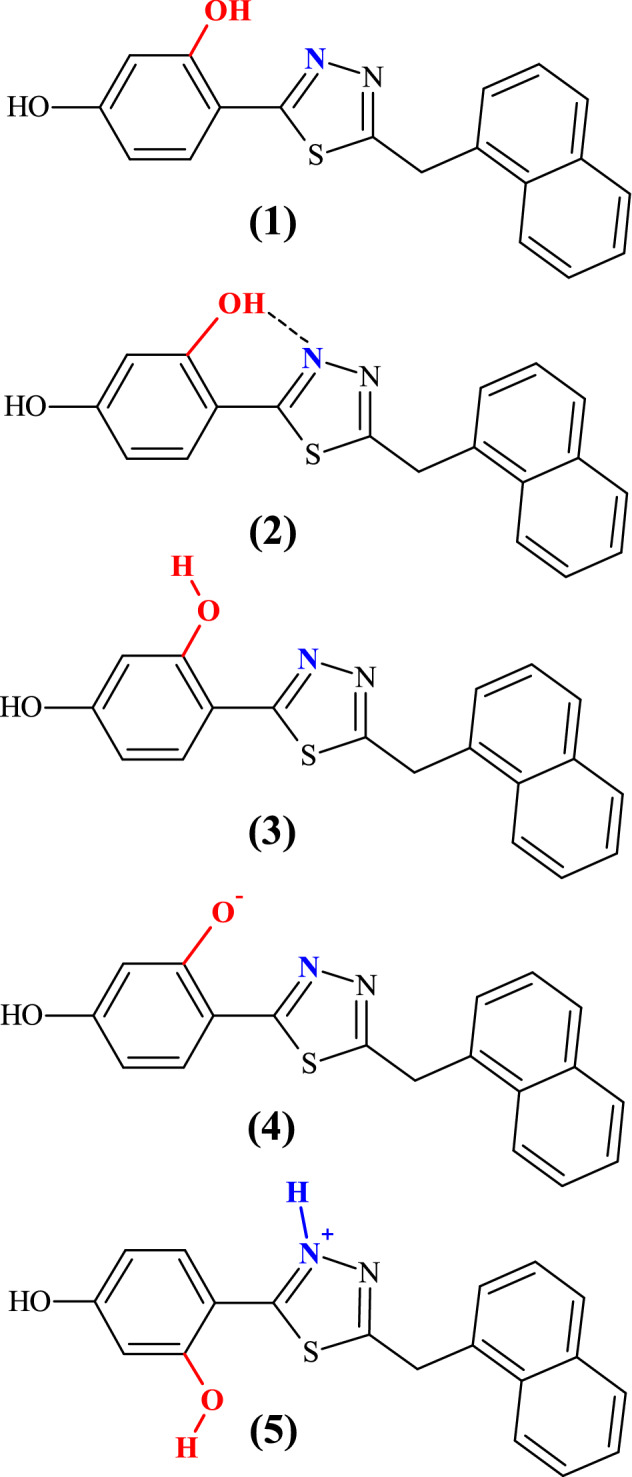
Chemical structure of 4-[5-(naphthalen-1-ylmethyl)-1,3,4-thiadiazol-2-yl]benzene-1,3-diol (NTBD) compound studied in this work (1) in its different structures: *cis-*enol form (2), *trans-*enol form (3), –O^-^ anion (4), and -N^+^-H cation (5).

The molecular spectroscopic studies described in this article were conducted in solvent systems and aqueous systems with variable pH on a notably interesting 1,3,4-thiadiazole-based molecule: *4-[5-(naphthalen-1-ylmethyl)-1,3,4-thiadiazol-2-yl]benzene-1,3-diol* (NTBD, Fig. 1). In particular, we carefully analyzed the molecular properties of NTBD in relation to the *enol → keto* ESIPT process. The effect, described for the first time in 1956 by Weller^23^ is both important and interesting given its growing applicability in various branches of science and industry, including new laser pigments^24^, solar cells^25^, OLED diodes^26^, polymer ultraviolet light^27^, molecular switches, molecular protein probes^28^, or in detection of trace amounts of DNA via FRET-based (Förster resonance energy transfer) methods^29^. Recent reports have also described a variation of the ESIPT phonomenon, specifically proton-transfer triggered proton transfer (PTTPT), in which one ESIPT process is induced by another ESIPT^30^. Pigments/molecules demonstrating *enol → keto* ESIPT share a number of interesting characteristics: (1) tautomerism usually takes place along a strong intramolecular hydrogen bond and is therefore very fast, which prevents or at least significantly limits non-radiative deexcitation of the excited *enol* form (enol*); (2) when significant structural reorganization takes place in the excited state, one can observe a massive Stokes shift that hinders reabsorption of the emitted light in the condensed phase; and (3) the quantum yield of fluorescence emission tends to increase in the solid state due to AIE effects, as the most efficient pathway of non-radiative deactivation of the excited keto* tautomer in the solution (rotation around the interring bond) is hindered, as in e.g. polymer matrices^31^. Furthermore, as follows from the latest reports, the ESIPT-related phenomenon of dual fluorescence is only observed in a very narrow energy window^32^.

It is known that organic chromophoric systems typically demonstrate diminished emission in aggregates or in the solid state as compared to their diluted solutions due to the aggregation-caused quenching (ACQ) effects^33^. However, Tang et al. observed a different emission properties of in a group of aromatic salts that showed weaker fluorescence in solutions and, uniquely, increased emission in the aggregated state. The effect was described as AIE^34^, and its mechanism was attributed to the restriction of intramolecular motion (RIM)^35,36^**.** A molecular rotation absorbs the excited-state energy via the non-radiative deactivation channel, whereas in the aggregated state, RIM can block the non-radiative channel and activate its radiative counterpart, consequently leading to relatively strong fluorescence emission^36^. Worldwide literature has since identified a number of different types of compounds capable of AIE [and the related aggregation-induced emission enhancement (AIEE)] fluorescence. Such systems can be utilized for the purposes of cellular imaging, as biosensors and fluorescence sensors, in optoelectronic and energetic devices, etc.^37^. With the above in mind, the aim of the study presented in this article was also to carefully evaluate an impact of aggregation effects via AIE on the (ESIPT-related) fluorescence properties for the NTBD molecule.

The article details comprehensive studies on NTBD conducted in several solvent media with varying polarities as well as selected mixtures (DMSO: H_2_O) and water solutions with different pH values employing UV–Vis absorption spectroscopy and fluorescence spectroscopy [including the technique of resonance light scattering (RLS)], as well as time-resolved fluorescence lifetime measurements in the frequency domain. The spectroscopic observations were subsequently complemented by the results of first principles density functional theory (DFT) and time-dependent DFT (TD-DFT) calculations and dipole moment estimations. Furthermore, the quantum yield values as well as radiative and non-radiative transition constants were determined for NTBD in solvents and mixed systems. The examined solvents were selected to strengthen the ESIPT effect and the related phenomenon of dual fluorescence, and to facilitate enhancement thereof by aggregation processes related to AIE. Finally, we also analyzed biological activity of NTBD relevant in the context of its antimycotic properties.

## Results and discussion

### Spectroscopic characterization of NTBD

The structure of the molecule selected for the study, *4-[5-(naphthalen-1-ylmethyl)-1,3,4-thiadiazol-2-yl]benzene-1,3-diol* (NTBD, Fig. 1), contains the characteristic resorcyl group connected with the 1,3,4-thiadiazole ring that in turn is linked via a –CH_2_ group to the naphthalene fragment. The latter is largely responsible for the molecule’s polarity as well as its phase separations^38,39^.

**Figure 2:**
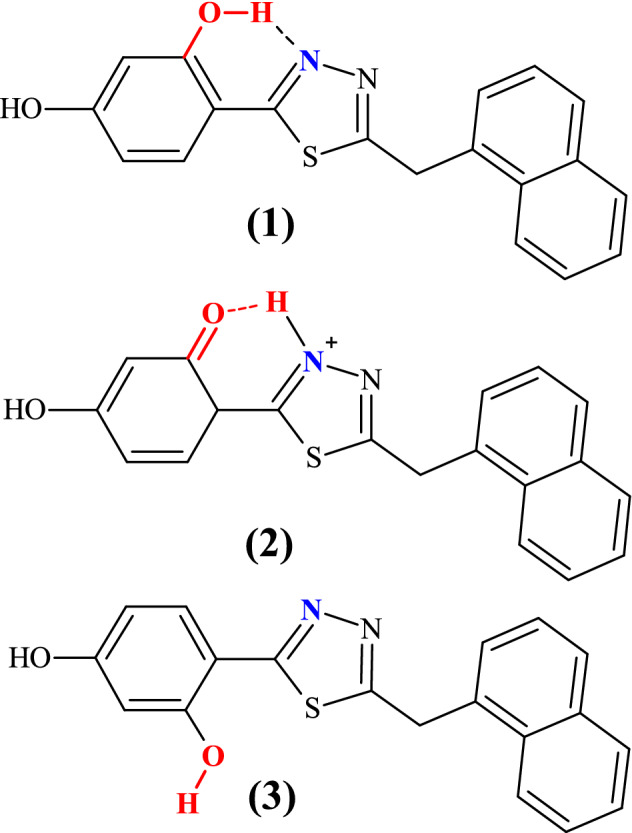
Keto-enol tautomerism and intramolecular hydrogen bonds in NTBD: *cis* configuration of enolic form (*cis*-enol) favoring the formation of intramolecular hydrogen bond with the nearest thiadiazole nitrogen atom (1), keto form (2) *trans* configuration of enolic form (*trans*-enol) in which the formation of intramolecular hydrogen bond is prevented (3).

**Figure 3:**
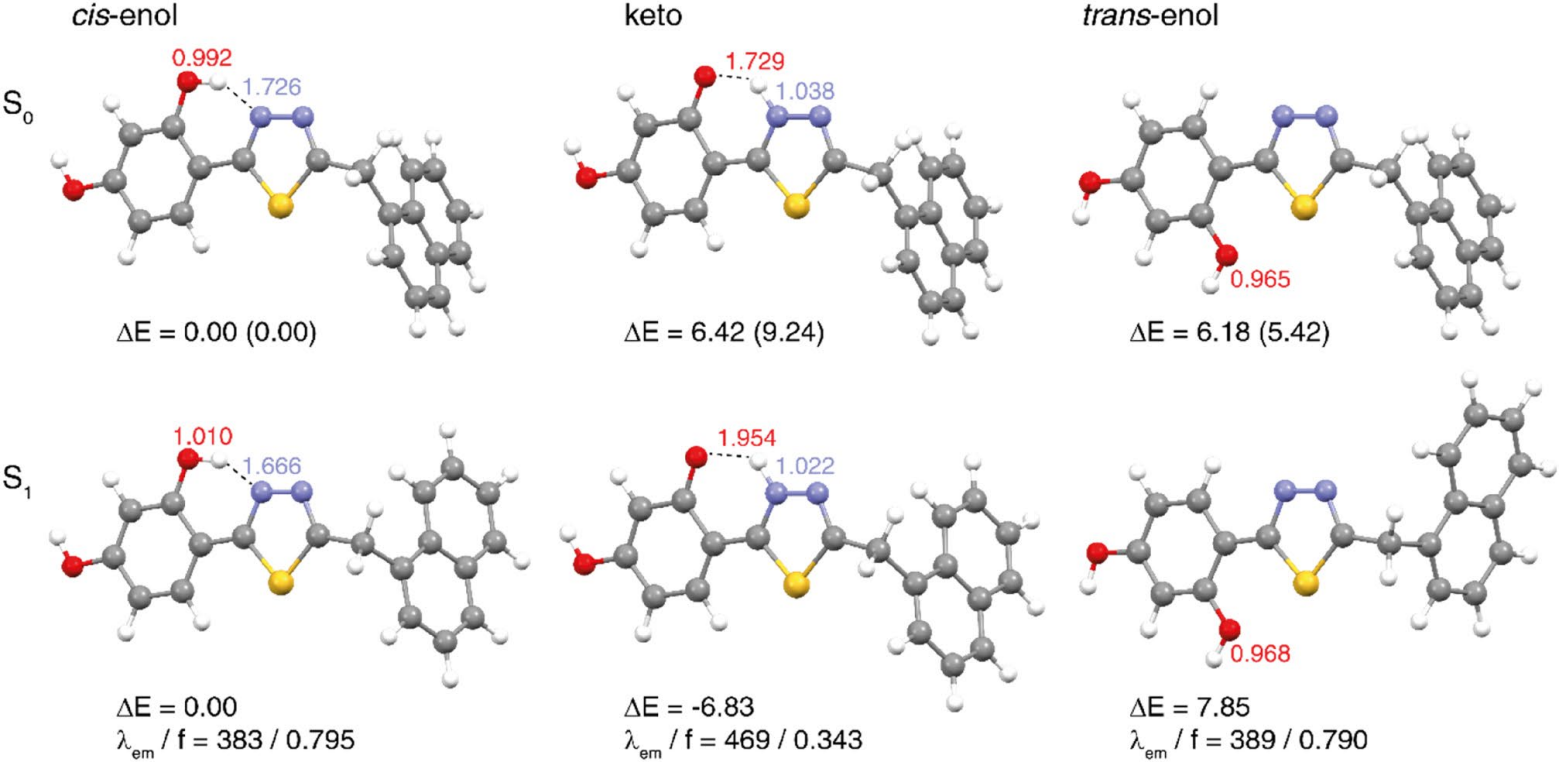
Optimized lowest-energy S_0_ ground-state (top, based on B3LYP+D3/6–311++G(d,p)/PCM(DMSO)) and the corresponding S_1_ excited-state (bottom, based on B3LYP/aug-cc-pVDZ/PCM(DMSO)) structures of enolic (*cis* and *trans*) and keto forms of NTBD. Values listed are the corresponding: interatomic distances *d*_OH_ (in red) and *d*_NH_ (in blue), in Å; relative energies Δ*E* computed with respect to the *cis*-enol structure, in kcal/mol, in parentheses Δ*E* values obtained with DSDPBEP86/6–311++G(d,p)/PCM(DMSO) at B3LYP + D3/6–311++G(d,p)/PCM(DMSO)-optimized structures are listed; fluorescence energies λ_em_, in nm, and oscillator strengths *f*. See also Tables S3 and S5.

As can be seen in Fig. 1, the examined system contains the hydroxyl group in the resorcyl group and the 1,3,4-thiadiazole nitrogen atom that may be located in close proximity, facilitating hydrogen-bond formation between these two sites and, accordingly, the *enol-keto* tautomerization. Indeed, as shown in the previous studies^40^, NTBD can exist in both *enol* and *keto* forms with the preference of the tautomeric structure depending on the solvent polarizability (with *enol* tautomer dominating in polar solvents and *keto* dominating in non-polar solvents with high average electric dipole moment polarizability). Furthermore, as follows from analyses on similar compounds, the –OH group in the resorcylic ring (*ortho* positions) can adopt two conformational structures^41^, namely, with hydrogen atom positioned either towards or away the *1,3,4-*thiadiazole moiety that corresponds to -OH located on the side of either the N atom or S atom of the *1,3,4-*thiadiazole (see Figs. 1 and 2)^40^. Only in the former structure (denoted here as *cis*-*enol*) the formation of the efficient intramolecular hydrogen bond is possible, along which the proton transfer can occur resulting in the ground- or excited-state *enol-keto* tautomerization. In the latter structure (denoted as *trans*-enol) the formation of the hydrogen bonding is precluded, preventing tautomerization processes. It is generally accepted that the *cis-*enol conformation is usually significantly more stable than its *trans-*enol counterpart. The aforementioned structural preferences for NTBD were indeed confirmed by the DFT calculations (B3LYP+D3/6–311++G(d,p) geometry optimizations with the continuum solvent model for DMSO, followed by the electronic energy evaluation employing the DSDPBEP86 double-hybrid density functional expected to provide more accurate results^42^; for a complete description of the computational details, see the Supplementary Materials). Namely, as shown in Fig. 3 and Table S3, although all three structures—*cis-*enol, *trans-*enol, and keto—were found to be stable in the ground state, the relatively high energy values obtained for *trans-*enol and keto indicate that their presence in DMSO solution is rather unlikely and that in such medium this compound probably exists preferentially in the *cis*-enol form.

**Figure 4:**
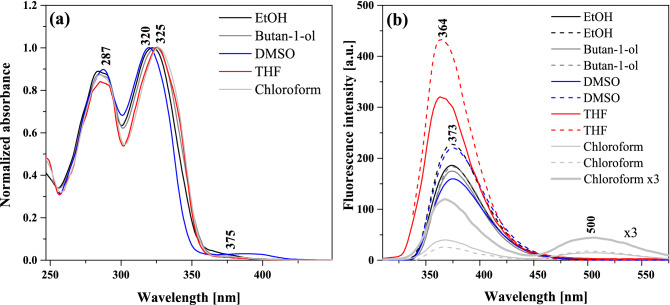
Normalized electronic absorption spectra (panel **a**) and fluorescence emission spectra (panel **b**) measured for NTBD in various solvents. The excitation wavelengths were 285 nm (solid line) and 325 nm (dotted line). Chloroform × 3 means the fluorescence intensity was multiplied by 3 for a better representation of the spectrum measured for NTBD in chloroform.

**Figure 5:**
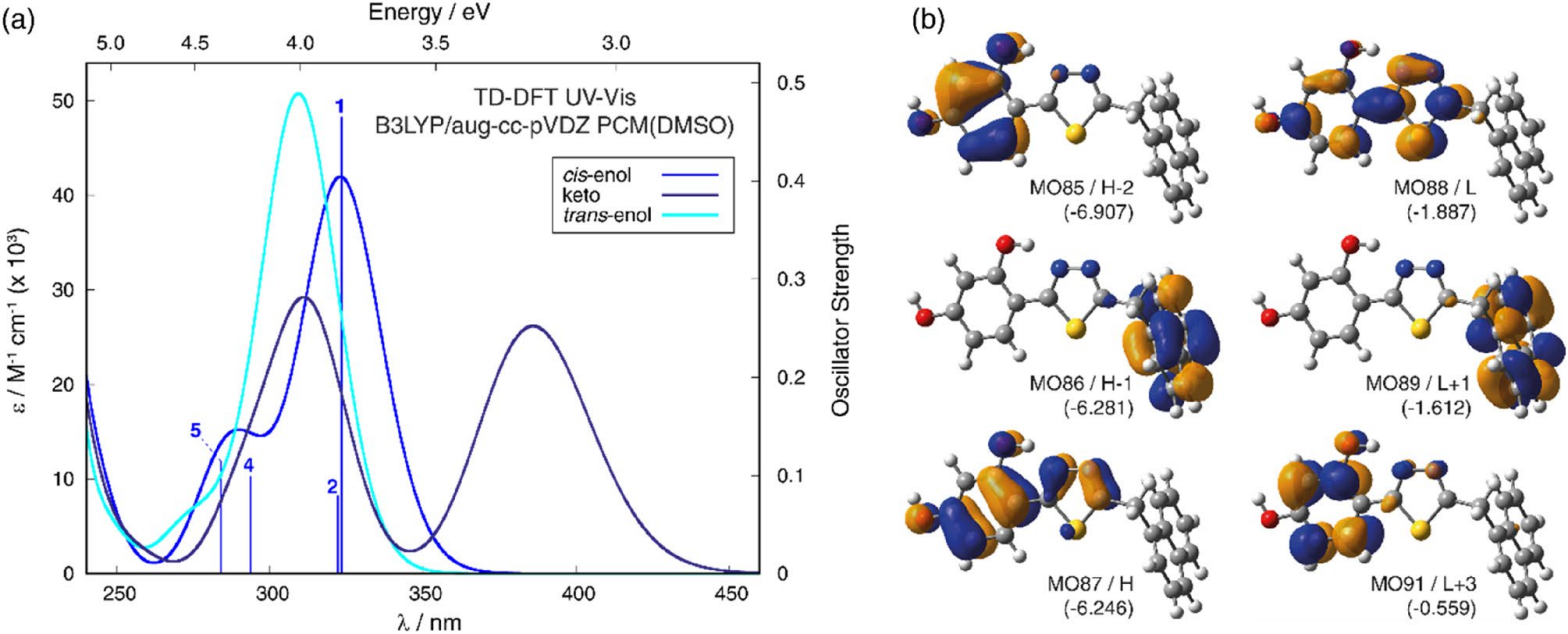
Panel (**a**): TD-DFT-simulated (B3LYP/aug-cc-pVDZ/PCM(DMSO) at B3LYP + D3/6–311 +  + G(d,p)/PCM(DMSO)-optimized structures) UV–Vis spectra for NTBD in its enolic (*cis* and *trans*) and keto forms with selected excitation energies and oscillator strengths calculated for *cis*-enol indicated as ‘stick’ spectra. Panel (**b**): Isosurfaces (± 0.04 au) of MOs involved in selected electronic transitions of NTBD *cis*-enol. H / L stands for HOMO/LUMO. Values listed in parentheses are corresponding orbital energies, in eV. For a full set of computed data, see Supplementary Materials.

Figure 4a presents the normalized electronic absorption spectra measured for NTBD in ethanol, butan-1-ol, DMSO, THF, and chloroform, i.e. solvents with significantly varied polarity characteristics. The positions of absorption maxima for other solvents examined in the study are presented in Table S1. As can be seen, with a change in the polarity of the medium, a slight but noticeable bathochromic shift was registered (Δλ = 5 nm (481 cm^-1^); Fig. 4a and Table S1) for the most intense band, respectively from 320 to 330 nm. The compound’s sensitivity to changes in the solvent polarity is therefore clearly observable. In the presented absorption spectra, we can note a wide lower-energy band within the range from 310 to 365 nm and with the maximum at ~ 320 nm, accompanied by the higher-energy band with the maximum at ~ 287 nm. Simultaneously, on the longwave side of the main signal, a slight additional maximum is present at ~ 370 nm. Its intensity and bathochromic shift evidence possible presence of aggregated forms of the compound (e.g. dimers or N-aggregates)^43^. As can be seen from Fig. 5, the TD-DFT-simulated (B3LYP/aug-cc-pVDZ with the continuum solvent model for DMSO) spectrum for the NTBD *cis*-enol structure agrees well with the experimental one, particularly in terms of the energetic positions of two main signals, confirming further the dominance of this form in DMSO solution. Furthermore, analysis of the computed dominant excitations for NTBD *cis*-enol shows that the lower-energy intense band observed experimentally around 320 nm originates mainly from S_0_ → S_1_ electronic excitation with the highest oscillator strength value, corresponding predominantly to HOMO → LUMO π–π * transition within the resorcylic-thiadiazole π-electron system, accompanied by less intense S_0_ → S_2_ excitation, assigned mainly to naphthalene → resorcylic-thiadiazole charge transfer (CT). The higher-energy band measured at around 287 nm appears to be due to S_0_ → S_4_ and S_0_ → S_5_ excitations that correspond predominantly to π-π* transition within the naphthalene fragment and CT-like π-π* within resorcylic-thiadiazole, respectively.

However, the most interesting effect observed in the course of the presented study is the one illustrated in Fig. 4b that shows the results of stationary fluorescence emission measurements for solvents for which the absorption spectra demonstrated in Fig. 4a were recorded. The data presented in both panels of Fig. 4 correspond to small concentrations of the analyzed compound in the examined solvent (~ 10^–5^ M). It is clearly visible in the figure that for most of the solvents—ethanol, butan-1-ol, DMSO, THF—only single fluorescence emission was present with the maximum at ~ 370 nm, and depending on their polarity, a slight shift of this band and a significant change in the intensity thereof was noticed. In other polar solvents, a single fluorescence emission band was also observed, as presented in Table S1. At the same time, for emission measured in chloroform, even regardless of the excitation wavelength, a very interesting effect of dual fluorescence appeared with the one maximum at ~ 370 nm, similar to other aforementioned solvents, accompanied by the second maximum at ~ 500 nm. The considerable Stokes shift of the longwave emission suggests a very significant change in the electronic structure of the emitting excited state^44^. Thus, when the polarity of the selected medium is decreased, the effect of dual fluorescence can be observed for NTBD. The dual fluorescence of NTBD in chloroform was significantly less intense as compared to the single fluorescence observed in polar solvents, which implies a considerably lower quantum yield of the emitting molecular form in the solvent system. The structural characteristics of NTBD, literature reports, and, primarily, presented below analysis of spectral data complemented by TD-DFT calculations all suggest that the observed for this compound emission effects, particularly dual fluorescence, are mainly due to excited-state intramolecular proton transfer (ESIPT) process. At a later stage of the study we were also able to demonstrate that in this case the ESIPT phenomenon was relatively easily enhanced by aggregation effects related to AIE, as evidenced by the induction of longwave emission by changes in the compound’s concentration or in the hydrophobicity of the medium.

**Figure 6:**
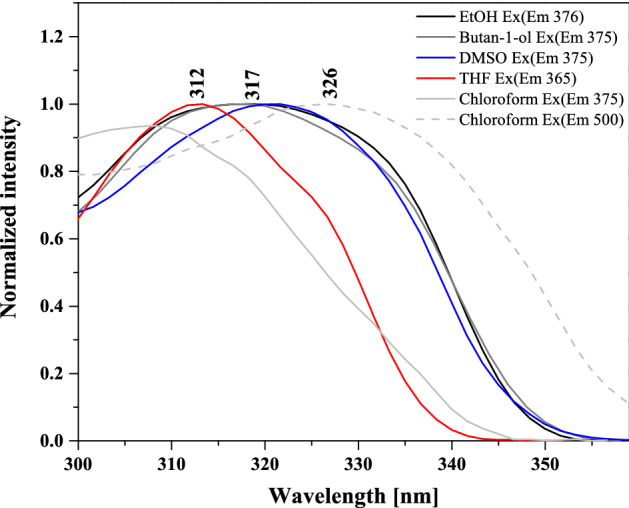
Normalized excitation spectra measured for NTBD in various solvents.

Fluorescence excitation spectra corresponding to the results presented in Fig. 4 were then also measured and they are demonstrated in Fig. 6. As aforementioned, in NTBD in its enolic form, the -OH group in the resorcylic ring can adopt either *cis* or *trans* conformation with respect to the N atom in the 1,3,4*-*thiadiazole ring and the positioning of the said group in one of these two extreme arrangements can either facilitate or prevent the proton transfer along the intramolecular –OH···N hydrogen bond. Namely, the *trans-*enol conformer lacks the relevant intramolecular hydrogen bonding and is therefore capable of producing only single fluorescence. In the *cis*-enol form, on the other hand, after photoexcitation, ultrafast excited-state proton transfer can occur along the hydrogen bonding. Namely, photoexcitation leads to a local increase in acidity and simultaneously alkalinity of the respective sections of the molecule that is directly due to the increased length of the O–H bond and the simultaneous shortening of the hydrogen bond N⋯H. Such effect indeed seems to be visible in the TD-DFT-optimized (B3LYP/aug-cc-pVDZ with the continuum solvent model for DMSO) S_1_ excited-state structure of NTBD *cis*-enol shown in Fig. 3; see also Table S5. Importantly, the calculations demonstrate that while both *cis*-enol and *trans*-enol structures upon excitation would emit at rather similar wavelength, nicely corresponding to the energetic position of the single fluorescence emission band and higher-energy band of the dual fluorescence signal, the latter demonstrates visibly higher energy, which combined with its higher energy also in the ground state, makes this form rather unlikely to be the dominantly emitting one. On the other hand, the computed S_1_ keto structure shows significantly lower energy and emits at visibly red-shifted wavelength relative to *cis*-enol, indicating that *enol → keto* tautomerization in the excited state, i.e. ESIPT, is thermodynamically allowed, and the co-existence of both tautomeric forms may accounts for the dual fluorescence effect observed for NTBD in chloroform solution. The lack of this effect in protic solvents might be due to the fact that intramolecular hydrogen bonds are very easily disrupted via intermolecular hydrogen bonds. Meanwhile, cleavage of intramolecular hydrogen bonds quickly reduces the population of *cis-*enol structure and leads to the formation of solvated enol while also significantly impacting the hydrophobicity of the medium—hence only a single fluorescence band is observed. At this point, it is very important to take a closer look at the excitation spectra presented in Fig. 6. A particularly interesting effect can be observed in the spectra registered for NTBD in chloroform after short and longwave excitation; the recorded spectra clearly show a shift relative to each other. This fact can be related to the presence of both *cis-* and *trans-*enol forms in the chloroform solution. The shift towards the longwave end of the excitation spectrum for *cis-*enol, as compared to *trans-*enol, is due to its inclusion of the mentioned intramolecular hydrogen bond. The results of [TD]DFT calculations confirmed the experimental results (Table S3). By combining the data presented in Fig. 4b for the emission spectra registered from the keto* form that showed a longwave shift relative to enol, with the excitation spectra of Fig. 6, we can conclude that the energy gap between the first exited state and the ground state was indeed lower in the keto* form. This suggests that, similarly to the emission spectrum, the absorption spectrum of the keto tautomer should also be shifted in the bathochromic direction relative to the enol absorption spectra that is indeed supported by the TD-DFT-simulated spectra for enol and keto structures of NTBD presented in Fig. 5.

**Figure 7:**
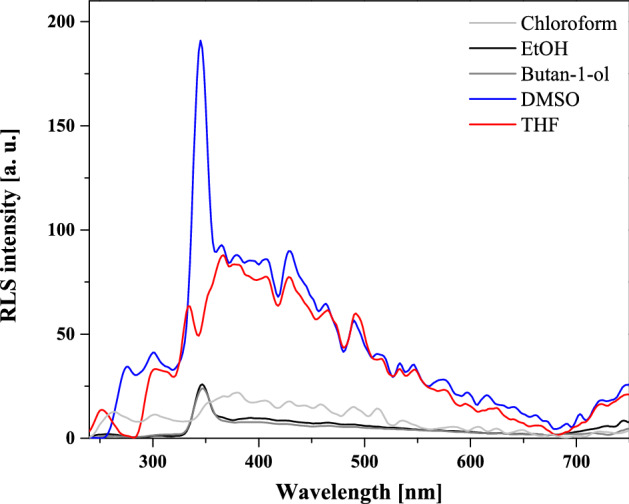
RLS spectra measured for NTBD in various solvents.

When analyzing the results presented in Figs. 4a and 6, it is noteworthy that in the case of the absorption spectra, even in polar solvents, a low-intensity longwave band was observed, while the corresponding excitation spectra were significantly widened. This may evidence the presence of aggregated forms of NTBD, such as dimers or larger aggregates. In order to confirm this conclusion, resonance light scattering (RLS) spectra corresponding to the spectra in Figs. 4 and 6 were measured for NTBD. As follows from literature data, in particular from papers by Pasternack and Colins^45^, detection of RLS spectra should be associated with the presence of aggregated forms of the compound in question. In our case, as shown in Fig. 7, RLS spectra were present predominantly in polar solvents with usually a single fluorescence emission. Nonetheless, even for synchronic spectra in chloroform, some bands, albeit of considerably lower intensity, were also detected. This confirms that indeed in certain solvents, effects related to aggregation may play a significant role in terms of changing the solvent’s local hydrophobicity and consequently facilitating the ESIPT process by reducing the isomerization energy barrier between the two tautomeric forms enol* and keto*, which will be elaborated in the subsequent part of this paper. Moreover, the very characteristic oscillatory structure of the registered RLS spectra indicates that in solution associated forms of NTBD varying in size potentially exist.

**Figure 8:**
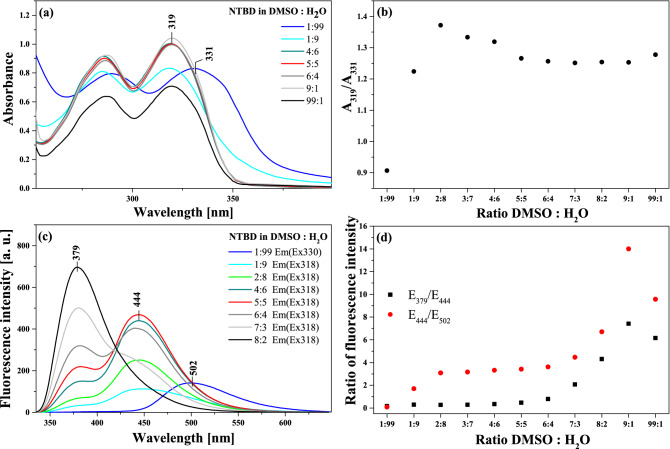
Panel (**a**): Absorption spectra of NTBD in DMSO: H_2_O solutions with various – 1:99, 1:9, 4:6, 5:5, 6:4, 9:1 and 99:1 – volume ratio. Panel (**b**): Correlation between the ratio of absorbance at the wavelength of 319 nm to the one at 331 nm and the ratio of DMSO to H_2_O in mixed solution. Panel (**c**): Fluorescence emission spectra of NTBD in DMSO: H_2_O solutions with various – 1:99, 1:9, 2:8, 4:6, 5:5, 6:4, 7:3 and 8:2 – volume ratio. The excitation was set at the absorbance maximum of each sample, respectively as pointed in the figure. Panel (**d**): Correlation between the ratio of fluorescence emission at the wavelength of 379 nm to the one at 444 nm along with the ones at 444 nm to 502 nm and the ratio of DMSO to H_2_O in mixed solution. The experiments were conducted at room temperature.

**Figure 9:**
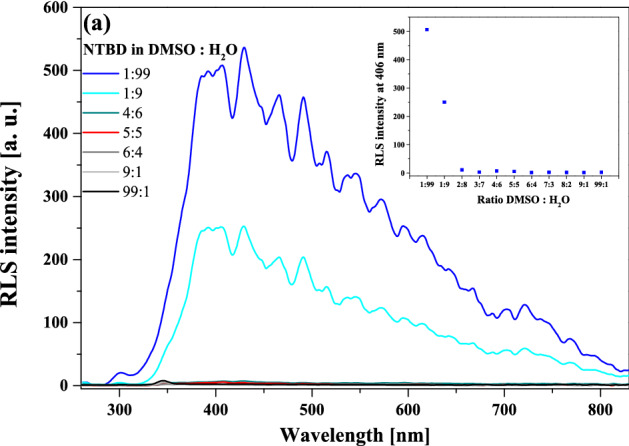
RLS spectra measured for NTBD in DMSO: H_2_O solutions with various – 1:99, 1:9, 4:6, 5:5, 6:4, 9:1 and 99:1 – volume ratio (panel a). The inset in (**a**) contains RLS spectra intensity at 406 nm versus DMSO: H_2_O solutions with various volume ratio.

Keeping in mind the results of the RLS measurements, at the subsequent stage of the study on the effects related to the ESIPT-induced phenomenon of dual fluorescence observed in NTBD molecules, we focused on the enhancement thereof by processes related to aggregation. In this context, Figs. 8 and 9 present the results confirming the aforementioned assumptions regarding the impact of aggregation on the analyzed ESIPT process and consequently the phenomenon of dual fluorescence. Figure 8a illustrates the electronic absorption spectra for NTBD in DMSO: H_2_O solutions with various volume ratio. As can be seen, even the addition of a small amount of water to DMSO resulted in a noticeable change in the intensity of the main absorption band with the maximum at ~ 320 nm. With the increasing amount of water in the mixture, the enhancement of the band on the longwave side became increasingly more visible, and at the ratio of 1:99 in favor of water, the lower-energy absorption band was very strongly shifted in the longwave direction, to 330 nm, and it was also significantly enhanced on the longwave side. As follows from Kasha’s exciton theory, the emergence of such effects in electronic absorption spectra clearly evidences the effects of chromophoric aggregation in the studied system; note that the exciton theory posits that longwave shifts correspond to the formation of *card pack* dimers^43^. Figure 8b presents the ratio of the absorbance measured at 319 nm to the one at 331 nm versus DMSO: H_2_O ratio, which reveals that the aggregation effects became visible in the solution relatively quickly. It is noteworthy that they became clearly noticeable at the mixture ratio of 1:9 in favor of water, and the maximum shift of the absorption spectrum was observed for the ratio of 1:99 in favor of water. At the same time, with the growing amount of water in the mixture, also the full width at half maximum of the absorption spectrum increased from 29 to 42 nm. However, an even more interesting effect is illustrated in Fig. 8c that presents the corresponding fluorescence emission spectra measured in DMSO: H_2_O mixed solutions. As can be seen, changing the ratio of DMSO: H_2_O, the effect of dual fluorescence for NTBD began to emerge with the maxima at 379 nm and 444 nm (interestingly, quite nicely corresponding to computed emission wavelength of S_1_ → S_0_ transitions for *cis*-enol and keto structures, see Fig. 3 and Table S5). As the water ratio increased, the intensity of the band with the maximum at 379 nm, corresponding to the enol* tautomer, decreased. At the same time, the band with the maximum at 444 nm retained its relatively high intensity for a surprisingly long time. At the ratio of 1:99, the these bands practically disappeared and a longwave band with the maximum at 500 nm emerged. The band at this wavelength corresponds to that previously registered for NTBD in chloroform and, as elaborated above, is characteristic of the excited form of the keto* tautomer. The discrepancies between the respective maxima are presented in Fig. 8d. These results clearly demonstrate that changes in the solvent’s polarity/hydrophobicity expedite the enol-keto transformation^46^. Namely, as follows from the data shown in Fig. 8c, the addition of water facilitates ESIPT in NTBD and accordingly the emission from the keto* form, which is significantly more intense than in the case of that observed in non-polar solvents due to aggregation effects. As evidenced in our previous research^41^, the aggregation of molecules of this type in water media is triggered by an increase in hydrophobic interactions. It is known that in solution, the molecules of the dissolved substance are surrounded by molecules of the solvent from all sides in three dimensions, but when single cells of the compound in question begin to aggregate, they are no longer completely surrounded by solvent molecules. Such aggregation significantly reduces accessibility of the solute to the solvent, which immediately reduces solvent–solute interactions in aggregates, increasing the hydrophobicity of the medium and consequently the population of the *cis-*enol form making it simultaneously more prone to ESIPT. The aggregation-induced increase in keto* tautomer emission has indeed already been described for several molecules capable of the ESIPT effect^47^ as aggregation-induced emission enhancement (AIEE)^48^, briefly mentioned above. The discussed results were further confirmed by measurements of the corresponding fluorescence excitation spectra for NTBD in DMSO: H_2_O mixed solutions, presented in Fig. S1 in the Supplementary Materials. The high selectiveness of fluorescence excitation spectra relative to absorption spectra provides an easy way to observe effects related to aggregation. It was revealed that already for a low amount of water added to DMSO, a significant decrease in the bands intensity was apparent, accompanied by the effects of bathochromic shift and considerable increase in the bands’ full width at half maximum. For the ratio of 1:99 water, the shift observed in the excitation spectra was very substantial, reaching ~ 340 nm. This was likely due to a combination of two effects, namely aggregation and the presence of both *cis-*enol and keto conformers.

To complement the analysis on the impact of aggregation effects related to AIE on excited-state proton transfer processes, Fig. 9 presents the RLS spectra for NTBD in DMSO: H_2_O mixed solutions. It is evident that the highest increase in the RLS spectra intensity was observed only at the ratios of 1:9 and 1:99 in favor of water, which further confirms the presence of strong aggregation effects. This is particularly well visible in the inset of Fig. 9 where changes in the RLS spectrum intensity with respect to the DMSO: H_2_O volume ratio are presented for the wavelength of 400 nm.

The NTBD molecule’s dipole moments were then computed using two methods. Fig. S2 as well as Tables 1 and S1 in the Supplementary Materials present the results obtained with the Bakhshiev, Kawski-Chamma-Viallet and Reichardt approach and with another similar method^49,50^. As shown above, NTBD produces dual fluorescence in non-polar solvents, see Fig. 4b for chloroform. The extensive Stokes shift visible for the longwave emission in such medium confirms a significant change in the electronic structure of the emitting excited state underlying this signal. Let us notice, however, that the relative intensity of the longwave fluorescence is comparable to that of the shortwave one; as follows from literature data, this intensity can change in response to an increased polarity of the medium—in non-polar but highly polarizable solvents, shortwave emission tends to outweigh longwave one. Furthermore, shortwave fluorescence undergoes a bathochromic shift as the polarity of the solvent grows along with its ability to form hydrogen bonds (Fig. 4b and Table S1). This means that polar solvents are capable to stabilize the excited state more than the ground state. As such, the bathochromic shift is caused by a reduction of the energy gap between those states. This confirms the fact that the excited state is more polar as compared to the corresponding ground state, which is a characteristic trait of emission originating from the keto* tautomer in molecules susceptible to the ESIPT effect. Further evidence supporting this point is also provided by our calculations of the mean dipole moment, the relevant results of which are provided in Fig. S2 as well as Tables 1 and S1. In the analyzed case, the calculations clearly evidence a change in the dipole moment averaged for all the solvents used. At the same time, as shown in Fig. S2b, the observed relationships are decidedly negatively inclined, which confirms that in the excited state, the dipole moment of the examined molecule may have a different sense than in the ground state, or indeed that the change thereof has a negative value. This shows that in the excited state, a strong reorganization of the electronic density distribution takes place, and in the specific case in question, that keto* tautomers are formed. This was indeed further supported by the results of the TD-DFT computations that revealed different electron density distribution in S_1_ *cis*-enol and keto structures as evidenced by the corresponding HOMO isosurfaces visualized in Fig. S11.

**Table 1:** Slopes m_1_ and m_2_ determined using respectively the plot of Stokes shifts against function F_1_(ε,n) and the plot of (${v}_{a}$ + ${v}_{f}$)/2 against ${\mathrm{F}}_{2}(\upvarepsilon ,\mathrm{ n})$.

	m_1_	m_2_	µ_e_/µ_g_	µ_e_ [D]	µ_g_ [D]	Δµ [D]	Number of points	r^2^
**Bakhshiev Kawski-Chamma-Viallet method**								
I	2016.44	− 960.53	2.82	3.14	1.11	2.03	8:7	0.83:0.58
I	3269.13	− 960.53	1.83	3.50	1.91	1.59	8:7	0.92:0.58
II	594.14	− 2145.42	1.77	5.32	3.01	2.31	6:8	0.84:0.82
II	4197.07	− 2145.42	3.09	4.63	1.50	3.14	8:8	0.92:0.82

	m	Δµ [D]	Number of points	r^2^				
**Reichardt method**								
I	1928.23	2.29	8	0.93				
II	2917.52	2.81	7	0.92				

Slope m was determined using the plot of Stokes shifts against function ${\mathrm{E}}_{\mathrm{T}}^{\mathrm{N}}$ along with ground-state dipole moment ${\upmu }_{\mathrm{g}}$, excited-state dipole moment ${\upmu }_{\mathrm{e}}$, change in dipole moment ∆μ, number of points and correlations factor r^2^ determined for both positions of maximum absorbance (I ~ 320 nm and II ~ 286 nm) for NTBD.

**Figure 10:**
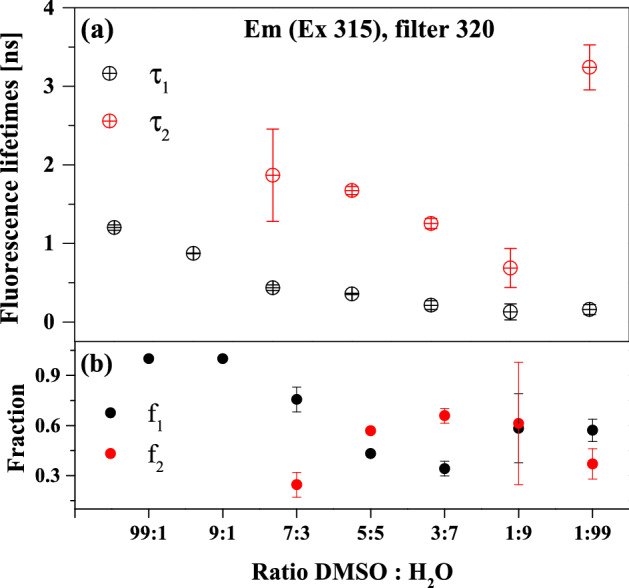
Fluorescence lifetimes (panel a), fractional intensities (panel b) and appropriate standard deviations measured for NTBD at various DMSO: H_2_O volume ratios using 320 nm filter.

Time-resolved measurements of fluorescence lifetimes in the frequency mode were also performed to further analyze the ESIPT phenomenon observed for NTBD and how it is influenced by aggregation effects. We focused primarily on the lifetimes in mixed DMSO: H_2_O systems; the corresponding results are presented in Figs. 10 and S3 as well as Table 2. As follows from the conducted measurements, two characteristic lifetimes could be identified in the mixed solutions. In cases where the emission spectra in Fig. 8 revealed only a single (higher-energy) fluorescence band characteristic for the excited enol form, we registered primarily the long-life component of ~ 1.4–2 ns. However, once the longwave band appeared in the fluorescence emission spectra, the lifetimes analysis started to return a clear two-component distribution. In the observations conducted with the 320 nm filter, the second component was found to be ~ 0.5 ns. Well-established literature data suggest that the shorter lifetime component is associated with the excited keto tautomer, while the longer lifetime one is related to the excited enol form. As such, the proportions where the enol* structures are dominant are characterized by primarily the longer fluorescence lifetime component, whereas, as soon as water begins to predominate in the medium, the lifetime fraction originating from the keto* form immediately emerges. As already discussed, the excitation spectra provide information mainly on the ground state, as the values registered at two different emission wavelengths were shifted relative to each other, confirming the fact that in both cases the emissions first decay towards different ground states. After the fluorescence emission, the keto tautomer quickly returns to the more preferred *cis*-enolic ground state, as evident in the electronic absorption spectra and supported by the quantum-chemical calculations (see discussion above).

**Table 2:** Fluorescence lifetimes τ [ns], fractional intensities f and standard deviation measured for NTBD at various DMSO: H_2_O volume ratio.

DMSO: H_2_O Em (Ex 315) filter 320	τ_1_ ± sd [ns]	f_1_ ± sd	τ_2_ ± sd [ns]	f_2_ ± sd
99:1	1.203 ± 0.033	1.000 ± 0.000		
9:1	0.873 ± 0.005	1.000 ± 0.000		
7:3	0.435 ± 0.035	0.756 ± 0.075	1.867 ± 0.588	0.245 ± 0.075
5:5	0.355 ± 0.009	0.432 ± 0.013	1.673 ± 0.050	0.568 ± 0.013
3:7	0.213 ± 0.055	0.342 ± 0.044	1.253 ± 0.064	0.658 ± 0.044
1:9	0.129 ± 0.102	0.583 ± 0.206	0.686 ± 0.250	0.612 ± 0.366
1:99	0.158 ± 0.063	0.571 ± 0.067	3.240 ± 0.287	0.370 ± 0.091

DMSO: H_2_O Em (Ex 331) filter 450	τ_1_ ± sd [ns]	f_1_ ± sd	τ_2_ ± sd [ns]	f_2_ ± sd
99:1	1.042 ± 0.159	1.000 ± 0.000		
9:1	0.115 ± 0.106	1.000 ± 0.000		
7:3	0.760 ± 0.239	0.372 ± 0.128	1.960 ± 0.221	0.612 ± 0.140
5:5	1.445 ± 0.035	1.000 ± 0.000		
3:7	1.146 ± 0.021	1.000 ± 0.000		
1:9	0.651 ± 0.013	1.000 ± 0.000		
1:99	0.441 ± 0.193	0.402 ± 0.076	2.721 ± 0.561	0.732 ± 0.216

The next part of the discussion pertains to calculations and analyses of the quantum yield of fluorescence Φ as well as radiative k_r_ and non-radiative k_nr_ decay constants for NTBD in various solvent systems, including DMSO: H_2_O mixtures. The calculations of said constants were naturally based on the data obtained from the aforementioned measurements of fluorescence lifetimes. The relevant results are shown in Fig. S4 as well as Tables 3 and S2. Figure S4a presents the NTBD quantum yield values obtained in EtOH, MeOH, DMF, THF, and chloroform, versus the solvent’s dielectric constant ε. As can be observed for the chloroform solution, wherein the ESIPT-induced dual emission was present, the quantum yield was significantly lower (~ 0.05) as compared to media in which single emission signal from the enol form of the molecule was primarily evident (~ 0.5–0.8). Fig. S4b demonstrates the changes of the quantum yield relative to the changes in the DMSO: H_2_O volume ratio in the NTBD mixed solutions. It is clear that the emission from the enol tautomer corresponds to increased fluorescence quantum yield. At the same time, as soon as the effect of dual fluorescence, particularly longwave fluorescence with the maximum at ~ 500 nm, appears, the value of Φ drops rapidly. Accordingly, it can be concluded that the fluorescence quantum yield of the enol tautomer is considerably higher than that of the keto form. It is also interesting to note the behavior of Φ for the mixture ratios in the range from ~ 3:7 to 7:3, where it stayed at an almost constant level, while, as shown by the emission spectra measurements, the effect of dual fluorescence was already present in the system, albeit with the respective maxima at ~ 380 and 440 nm (Fig. 8c). What follows from these results is that the fluorescence quantum yield was overall higher in polar as compared to non-polar solvents, and that aggregation triggers a significant decrease thereof. We also know that the tautomeric equilibrium must also be present in polar solvents, but in order to observe the ESIPT effect in such media, it is necessary to include the additional factor of changing medium hydrophobicity, e.g. due to aggregation effects related to AIE fluorescence.

**Table 3:** Rate constants of radiative (*k*_r_) and non-radiative (*k*_nr_) intramolecular processes for NTBD in various solvents and mixed DMSO: H_2_O systems.

DMSO: H_2_O	τ_1_	${\Phi }_{F}$	k_r_ [ns^-1^]	k_r_ [s^-1^]	k_nr_ [ns^-1^]	k_nr_ [s^-1^]
99:1	1.203	0.3735	0.31	3.10‧10^–10^	0.52	5.21‧10^–10^
9:1	0.873	0.2594	0.30	2.97‧10^–10^	0.85	8.48‧10^–10^
7:3	0.435	0.179	0.41	4.11‧10^–10^	1.89	1.89‧10^–9^
5:5	0.355	0.1936	0.55	5.45‧10^–10^	2.27	2.27‧10^–9^
3:7	0.213	0.1308	0.61	6.14‧10^–10^	4.08	4.08‧10^–9^
1:9	0.129	0.0573	0.44	4.44‧10^–10^	7.31	7.31‧10^–9^
1:99	0.158	0.0265	0.17	1.68‧10^–10^	6.16	6.16‧10^–9^

Solvent	τ_1_	${\Phi }_{F}$	k_r_ [ns^-1^]	k_r_ [s^-1^]	k_nr_ [ns^-1^]	k_nr_ [s^-1^]
Methanol	1.72	0.82	0.48	4.78‧10^–10^	0.10	1.03‧10^–10^
Ethanol	1.76	0.73	0.42	4.16‧10^–10^	0.15	1.53‧10^–10^
DMF	1.53	0.48	0.31	3.12‧10^–10^	0.34	3.42‧10^–10^
THF	1.61	0.13	0.08	7.99‧10^–11^	0.54	5.41‧10^–10^

Very interesting properties of can also be observed for the corresponding radiative k_r_ and non-radiative k_nr_ transition constants in NTBD, presented in Table 3. Notably, with the decreasing polarity of the solvent, the value of the radiative constant quickly decreased, while the non-radiative constant was significantly increased. Similar results were obtained in the DMSO: H_2_O solvent mixtures. Namely, the k_r_ value first slightly increased, but once the DMSO: H_2_O ratio reached 1:9 or 1:99 in favor of water, it dropped drastically. The value of k_nr_ increased significantly for the ratios from 0.52 to 6.16. Overall, the results confirmed that with the increasing medium inclination towards aggregation and corresponding changes in its hydrophobicity, non-radiative deactivation of the NTBD molecule’s excitation becomes more efficient, which also indicates an increase in the preference of the keto tautomer in the excited state. Such behavior is fairly characteristic of molecules capable of ESIPT in solvent systems. The corresponding behavior in the solid state systems will be explored in our future studies.

**Figure 11:**
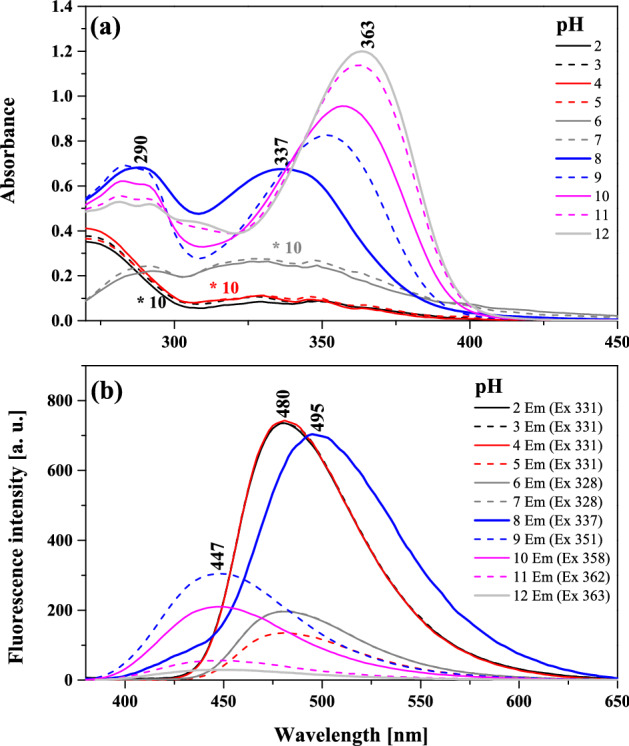
Absorption spectra for NTBD in water solution with varying pH (from 2 to 12) (panel **a**) along with the corresponding fluorescence emission spectra (panel **b**). The spectra were measured at room temperature.

Finally, we aimed to determine what processes related to fluctuations in pH of water solution can affect the analyzed changes in the fluorescence emission spectra for NTBD. The relevant results are provided in Figs. 11, S5, and S6 in the Supplementary Materials. As follows from the data in Fig. 11a, the ionization of the –OH group in the resorcylic ring caused a bathochromic shift of the absorption band maximum to 363 nm (27.548 cm^-1^) at pH = 12, relative to the maximum of 337 nm (29.673 cm^-1^) at pH = 7.5. The Δλ between the spectra was in this case 26 nm (i.e. 2125 cm^-1^). As the pH continued to decrease, we observed a shift of the absorption band maximum to 325 nm (30.769 cm^-1^); based on our earlier studies on similar topics and crystallographic data obtained for analogous 1,3,4*-*thiadiazoles^41^, at low pH levels ionic forms with the ionized -N^+^-H group become predominant. Moreover, spectra recorded at low pH show a characteristic enhancement on the longwave side with the maximum at ~ 360 nm (27.777 cm^-1^). Said changes are particularly visible in the fluorescence excitation spectra presented in Fig. S5 in the Supplementary Materials. As follows from Kasha’s exciton theory^51^, effects of this type are related to the possibility of *card pack* molecular aggregation in the given medium. By employing the molecular distance formula, said theory allowed us to calculate the distance between the chromophores in the aggregate state as R_β_ = 3.45 Å, which is in the range of the DFT and crystallographic data found for analogous systems^41^. Figure 11b presents the corresponding results for the emission spectra measurements. As can be seen, for the fluorescence emission spectrum originating from the ionized form of NTBD with the -O^-^ group the maximum was recorded at ~ 447 nm, while for the form with the -N^+^-H group it was shifted to ~ 480 nm with a simultaneous significant increase in intensity. The corresponding absorption spectra show that in this particular case, processes related to aggregation were dominant, as the absorption spectrum was considerably decreased. At this point, it is worth referring back to the results presented in Fig. 8 and the fact that in the initial phase of the experiment with the increase in the water content in the DMSO: H_2_O mixture, NTBD demonstrated dual fluorescence with the second (lower-energy) maximum at ~ 440 nm. It was only in the subsequent stages of the measurements that the longwave band with the maximum at ~ 500 nm appeared. This corroborates the conclusion that the emission spectrum with the maximum at ~ 440 nm might be associated with the ionized form of the NTBD molecule with the –O^-^ group. Notably, at the pH range from 7.5 to 8.5, we observed a slight dual fluorescence effect with the maxima at ~ 420 and 500 nm. The fact that this was only observed in such a narrow pH window tells us that the ionic forms of this particular molecule exert a strong influence on stationary fluorescence spectra. The impact of ionized forms on aggregation-related effects is clearly visible in Fig. S6 that shows the corresponding RLS spectra. As can be observed in the pH range from 2 to 5, the RLS signal at the maximum had the intensity of ~ 200 a.u., which evidenced fairly significant aggregation effects. However, at the physiological pH range of 6–8, the signal suddenly increased to ~ 450 a.u., which indicated enhancement of aggregation impact on the fluorescence emission spectra, while a slight dual fluorescence effect was observed practically only at this range as well. At high pH ≥ 9, the RLS signal decreased to ~ 120 a.u. This suggests a likely increase in the number of NTBD molecules containing the ionized –O^-^ group and strong monomerization of the medium at this pH. The emission spectra revealed an increase in intensity of the band with the maximum at ~ 440 nm, which is consistent with only a handful of reports available in literature^52,53^.

### Antimicrobial properties of NTBD

According to criteria of bioactivity proposed by O’Donnell et al.^54^, the examined compound revealed no significant bioactivity neither on the tested Gram-negative and Gram-positive reference bacterial strains (MIC > 1000 mg/L, Table 4). However, it demonstrated good antifungal activity (MIC in the range 26–125 mg/L) against *C. albicans* and *C. parapsilosis* strains and moderate bioactivity (MIC in range 126–500 mg/l) against *C. glabrata* strain^54^. Vancomycin (Van), and nystatin (Nys) were used as the standard drugs. There were no differences in MIC values of NTBD samples dissolved in the DMSO: H_2_O mixtures with the increasing water content.

**Table 4:** Antibacterial and antifungal activities of NTBD presented in minimal inhibitory concentration (MIC) values.

Chemicals Microorganism	MIC (mg/l)	
Gram-positive bacteria	NTBD	Van
*S. aureus* ATCC 25923	2500	0.98
*S. epidermidis* ATCC 12228	2500	0.98
*M. luteus* ATCC 10240	31,3	0.12
*B. subtilis* ATCC 6633	1250	0.24
*B. cereus* ATCC 10876	1250	0.98
*E. faecalis* ATCC 29212	1250	1.95

Gram-negative bacteria		Cip
*S. typhimurium* ATCC 14028	1250	0.061
*E. coli* ATCC 25922	2500	0.015
*P. mirabilis* ATCC 12453	2500	0.030
*K. pneumoniae* ATCC 13883	2500	0.122
*P. aeruginosa* ATCC 9027	1250	0.488

Yeasts		Nys
*C. glabrata* ATCC 90030	250	0.24
*C. albicans* ATCC 102231	62.5	0.48
*C. parapsilosis* ATCC 22019	31.3	0.24

*Van* vancomycin, *Cip* ciprofloxacin, *Nys* nystatin.

## Conclusions

The presented results of spectroscopic studies supported by dipole moment calculations and (TD-)DFT quantum-chemical modeling provided comprehensive evidence that the effect of dual fluorescence observed in the spectra of the NTBD molecule is related to the phenomenon of *cis*-enol → keto excited-state intramolecular proton transfer (ESIPT) with the first (higher-energy) emission band in the emission spectrum originating from the excited enol and the second (lower-energy) one from the excited keto form. However, in DMSO: H_2_O mixed media where significant hydrophobicity changes facilitate interactions between 1,3,4-thiadiazole molecules, the dual fluorescence effects in NTBD were strongly influenced by the phenomenon of aggregation related to AIE fluorescence. Aggregation weakens strong solvent–solute interactions, thus facilitating the tautomerization process in the excited state, and consequently appearance of dual fluorescence signal. The relevant quantum yield calculations revealed that the keto* tautomer’s yield is lower than that of enol*, and the effect was further strengthened by aggregation. Furthermore, the calculations of radiative and non-radiative transition constants showed that the deactivation of the excited state was strongly affected by the discussed aggregation-related effects that visibly enhanced the non-radiative deactivation channels. The measurements of the concentrations of hydrogen ions also confirmed the presence of ionized forms of the NTBD molecule containing the –O^-^ and –N^+^–H groups, which significantly affected the observed fluorescence emission spectra as they produced a relatively strong emission that overlapped with the effects related to the ESIPT process. Finally, we showed that NTBD revealed good antifungal activity and may be considered as promising anti-yeast agent.

## Materials and methods

### Materials

4-[5-(Naphthalen-1-ylmethyl)-1,3,4-thiadiazol-2-yl]benzene-1,3-diol (NTBD) was synthesized in the Department of Chemistry of the University of Life Sciences in Lublin and described in the previous publication^40^.

Stock solutions of NTBD were prepared by dissolving approximately 1 mg of the compound in selected solvents (methanol, ethanol, butan-1-ol, propan-2-ol, acetonitrile, ethyl acetate, DMSO, DMF, THF, acetone, toluene, chloroform, *n-*hexane and *n-*heptane). All the solvents used in this study were of analytical grade. An appropriate volume of the solution was added to 2 mL of a given solvent in order to obtain the required absorbance intensity. The molar concentrations of NTBD dissolved in THF and DMSO were respectively 1.49·10^–3^ M and 1.35·10^–3^ M.

### Methods

#### Electronic absorption and fluorescence spectra

Electronic absorption spectra of NTBD were recorded on a double-beam UV–Vis spectrophotometer Cary 300 Bio (Varian) equipped with a thermostated tray holder with a 6 × 6 multi-cell Peltier block. The temperature was controlled with a thermocouple probe (Cary Series II from Varian) placed directly in the sample.

Cary Eclipse spectrofluorometer (Varian) was applied for recording fluorescence excitation, emission and synchronous spectra. All measurements were carried out at 22 °C. All the fluorescence spectra were recorded with 0.5 nm resolution together with the lamp and photomultiplier spectral characteristics corrections. Resonance light scattering (RLS) measurements were carried out according to the previously reported protocol with synchronous scanning of both the excitation and emission monochromators (there was no interval between excitation and emission wavelengths) and spectral resolution of 1.5 nm. Grams/AI 8.0 software (Thermo Electron Corporation; Waltham, Massachusetts, United States) was applied for an analysis of the recorded data.

#### Estimation of dipole moment values

Ground- and excited-state dipole moment values of the NTBD molecule were estimated employing two methods based on the influence of the internal electric field (solvatochromism), i.e. the impact of the solvents on the locations of the maxima of absorption and fluorescence spectra of the system in question.

#### Method 1

Based on the papers by Bakshiev and Kawski-Chamma-Viallet^49,50^, the change in the dipole moment between the ground and excited state can be calculated from the following two Eqs. (1) and (2):

1 $$ \overline{v}_{a} - \overline{v}_{f} = m_{1} F_{1} \left( {\varepsilon ,n} \right) + cste $$

2 $$ \frac{{\overline{v}_{a} + \overline{v}_{f} }}{2} = m_{2} F_{2} \left( {\varepsilon ,n} \right) + cste $$

where $\overline{v}_{a}$ and $\overline{v}_{f}$ are the absorption and fluorescence maxima expressed in cm^-1^. The expressions $F_{1} \left( {\varepsilon ,n} \right)$ (Bakshiev’s polarity function) and $F_{2} \left( {\varepsilon ,n} \right)$ (Kawski-Chamma-Viallet function) for a given solvent are presented as Eqs. (3) and (4):

3 $$ F_{1} \left( {\varepsilon ,n} \right) = \frac{{2n^{2} + 1}}{{n^{2} + 2}} \cdot \left( {\frac{\varepsilon - 1}{{\varepsilon + 2}} - \frac{{n^{2} - 1}}{{n^{2} + 2}}} \right) $$

4 $$ F_{1} \left( {\varepsilon ,n} \right) = \frac{{2n^{2} + 1}}{{2\left( {n^{2} + 2} \right)}} \cdot \left( {\frac{\varepsilon - 1}{{\varepsilon + 2}} - \frac{{n^{2} - 1}}{{n^{2} + 2}}} \right) + \frac{{\left( {n^{4} - 1} \right)}}{{2(n^{2} + 2)^{2} }} $$

where $n$ is the light refraction index and $\varepsilon$ is the solvent’s dielectric constant. The dipole moment change is calculated based on the locations of absorbance and fluorescence maxima for the given molecule in various solvents. Assuming that the symmetry of the analyzed molecule remains unchanged following the electronic transition, and that the ground- and excited-state dipole moments are parallel, we obtain the following Eqs. (5) and (6):

5 $$ \mu_{g} = \frac{{\left| {m_{1} + m_{2} } \right|}}{2} \cdot \left( {\frac{{hca_{0}^{3} }}{{2m_{1} }}} \right)^{1/2} $$

6 $$ \mu_{e} = \frac{{\left| {m_{1} - m_{2} } \right|}}{2} \cdot \left( {\frac{{hca_{0}^{3} }}{{2m_{1} }}} \right)^{1/2} $$

where $\mu_{g}$ and $\mu_{e}$ are the ground and exited-state dipole moments, respectively, $h$ is Planck’s constant, $c$ is the velocity of light, and $a_{0}$ is the radius of the molecule’s Onsager cavity that is calculated from the Suppan’s Eq.  (7)^55^:

7 $$ a_{0} = \left( {\frac{3M}{{4\pi \delta N}}} \right)^{\frac{1}{3}} $$

with $\delta$ being the density of the dissolved substance, $M$ being its molar mass, and $N$ being the Avogadro constant. The ratio of the excited-state dipole moment to the ground-state dipole moment can be expressed as (8):

8 $$ \frac{{\mu_{e} }}{{\mu_{g} }} = \frac{{\left| {m_{1} - m_{2} } \right|}}{{\left| {m_{1} + m_{2} } \right|}} $$

where $m_{1}$ and $m_{2}$ are the respective slopes of straight lines obtained from the graphs representing the Stokes shifts relative to the solvent polarity function $F_{1} \left( {\varepsilon ,n} \right)$ and $\left( {\overline{v}_{a} + \overline{v}_{f} } \right)/2$ relative to the function $F_{2} \left( {\varepsilon ,n} \right)$. The change in the molecule’s dipole moment is determined as the difference between the excited-state dipole moment and the ground-state dipole moment [Eq. (9)]:

9 $$ \Delta \mu = \mu_{e} - \mu_{g} . $$

#### Method 2

The second method, first proposed by Reichardt^56^, utilizes a scale describing the microscopic polarity of solvents $E_{T}^{N}$ assessed based on the solvatochromic effect present in the betaine pigment. The polarization dependence and presence of hydrogen bonds in solvents are correlated with the polarity scale $E_{T}^{N}$. The theoretical basis for the correlation between the spectral shift and the $E_{T}^{N}$ scale was provided by Ravi et al.^57^.The $E_{T}^{N}$ value is defined by Eq. (10), where water ($E_{T}^{N} = 1$) and tetramethylsilane ($E_{T}^{N} = 0$) are used as the reference solvents:

10 $$ E_{T}^{N} = \frac{{E_{T} \left( {solvent} \right) - E_{T} \left( {TMS} \right)}}{{E_{T} \left( {water} \right) - E_{T} \left( {TMS} \right)}} = \frac{{E_{T} \left( {solvent} \right) - 30.7}}{32.4} $$

Finally, the molecular dipole moment change $\Delta \mu$ can be expressed via Eq. (11):

11 $$ \Delta \mu = \mu_{e} - \mu_{g} = \sqrt {\frac{m \cdot 81}{{11307.6 \cdot \left( {\frac{6.2}{{a_{0} }}} \right)^{3} }}} $$

where $m$ is the slope of the straight line obtained from the graph of the Stokes shifts relative to the parameter $E_{T}^{N}$.

#### Fluorescence quantum yields

Quantum yields of fluorescence of NTBD solutions were determined using 7-diethylamino-4-methylcoumarin (coumarin1) as the fluorescence standard. The measurements were carried out in ethanol ${\Phi }_{F} = 0.73$^58^. The final fluorescence quantum yield values were calculated based on Eq. (12):

12 $$ {\Phi }_{F\left( X \right)} = {\Phi }_{{R\left( {MeOH} \right)}} \left( {\frac{{\lambda_{{exR\left( {EtOH} \right)}} }}{{\lambda_{exX} }}} \right)\left( {\frac{{I_{X} }}{{I_{{R\left( {EtOH} \right)}} }}} \right)\left( {\frac{{\eta_{X}^{2} }}{{\eta_{{R\left( {EtOH} \right)}}^{2} }}} \right) $$

where the subscript $X$ denotes NTBD in various solvents and mixed systems and the subscript $R\left( {EtOH} \right)$ denotes coumarin1 in EtOH solution, $\lambda_{ex}$ is the value of absorbance at the excitation wavelength, $I$ is the area under the emission curve, and $\eta$ is the refractive index of the solvent.

#### Rate constants of radiative (*k*_r_) and non-radiative (*k*_nr_) intramolecular processes

Quantum yield (Φ) is defined as the ratio of the number of photons emitted to the number of photons absorbed and linked with radiative and non-radiative process rate constants via Eq. (13)^59^:

13 $$ {\Phi } = \frac{{k_{r} }}{{k_{nr} + k_{r} }} = \frac{number of photon emitted}{{number of photon absorbed}} $$

where $k_{r}$ is the rate constants for radiative process, while $k_{nr}$ is the rate constants for non-radiative process.

The measured lifetime of fluorescence ($\tau$) can be related to the rate constants for radiative and non-radiative decay $k_{r}$ and $k_{nr}$ as presented in Eq. (14):

14 $$ \tau = \frac{1}{{k_{r} + k_{nr} }} $$

Consequently, the basic photophysical equations which correlate the quantum yield and lifetime of fluorescence of the molecule with rate constants of radiative and non-radiative processes can be defined as follows^60^:

15 $$ k_{r} = \frac{{{\Phi }_{F} }}{\tau } $$

16 $$ k_{nr} = \frac{{1 - {\Phi }_{F} }}{\tau } $$

where in our case ${\Phi }_{F}$ and $\tau$ is respectively the fluorescence quantum yield and fluorescence lifetime measured for NTBD solution.

#### Fluorescence lifetimes

Fluorescence lifetimes were measured with a frequency domain method, using a multifrequency cross-correlation phase and modulation K2 fluorometer (ISS, Champaign, IL). NTBD was dissolved in organic solvents and in DMSO: H_2_O mixtures. Fluorescence emission was recorded in 10 × 10 mm (or 4 × 4 mm for high NTBD concentration) quartz cuvette, for the excitation at 315 nm (300 W xenon arc lamp), through a cut-off filter (transmittance for λ > 320 nm) in the emission channel. Measurements were performed for 15–20 modulation frequencies ranging from 2 to 200 MHz, using water solution of Ludox@ (Aldrich, Darmstad, Germany) as a reference. Data were analyzed according to a multiexponential decay model for discrete fluorescence lifetime components with the use of Vinci 2.0 software supplied by the ISS Company:

$$ I\left( {\lambda ,t} \right) = \mathop \sum \limits_{i} \frac{{f_{i } (\lambda )}}{{_{i} }}e^{{ - {\raise0.7ex\hbox{$t$} \!\mathord{\left/ {\vphantom {t {_{i} }}}\right.\kern-0pt} \!\lower0.7ex\hbox{${_{i} }$}}}} $$

where $I\left( {\lambda ,t} \right)$ is fluorescence intensity and $f_{i }$ is fractional contribution of each fluorescence lifetime component.

Best-fit parameters were obtained by minimising both the reduced χ2 value and the residual distribution of the experimental data. Measurements were repeated 3–5 times and the average values with standard deviations were calculated.

#### Antimicrobial properties

Antibacterial and antifungal activities of NTBD was tested by microdilution broth method according to the European Committee on Antimicrobial Susceptibility Testing (EUCAST) (www.eucast.org) using Mueller–Hinton broth or RPMI with MOPS for growth of fungi by methods described elsewhere^61^. Minimal Inhibitory Concentration (MIC) of the compound was evaluated for the panel of the reference microorganisms from American Type Culture Collection (ATCC), including Gram-negative bacteria (*Escherichia coli* ATCC 25922, *Salmonella Typhimurium* ATCC14028, *Klebsiella pneumoniae* ATCC 13883, *Pseudomonas aeruginosa* ATCC 9027, *Proteus mirabilis* ATCC 12453), Gram-positive bacteria (*Staphylococcus aureus* ATCC 25923, *Staphylococcus epidermidis* ATCC 12228, *Micrococcus luteus* ATCC 10240, *Enterococcus faecalis* ATCC 29212, *Bacillus subtilis* ATCC 6633, *Bacillus cereus* ATCC 10876), and fungi (*Candida albicans* ATCC 10231, *Candida parapsilosis* ATCC 22019, *Candida glabrata* ATCC 90030).

#### Quantum–mechanical calculations

All computations were performed at the density functional theory (DFT) and its time-dependent variant approach with continuum solvent model for DMSO. A full description of computational details used in these studies along with appropriate references is provided in the Supplementary Materials.

## Supplementary Information

Supplementary Information.

## Data Availability

The datasets used and/or analysed during the current study available from the corresponding author on reasonable request.
