# Supplementary material for: Spectroscopic characterization and assessment of microbiological potential of 1,3,4-thiadiazole derivative showing ESIPT dual fluorescence enhanced by aggregation effects
Source: Sci Rep. 2022 Dec 22;12:22140. doi: 10.1038/s41598-022-26690-1 (PMC9780306; doi:10.1038/s41598-022-26690-1)
Supplement: Supplementary file 1 — Supplementary Information. [file 41598_2022_26690_MOESM1_ESM.docx]

**SUPPLEMENTARY MATERIALS**

**Additional experimental data**

**Figure S1.** Fluorescence excitation spectra of NTBD in DMSO : H_2_O solutions with various volume ratio. The figure presents spectra for ratio 1:99, 1:9, 2:8, 4:6, 5:5, 6:4, 7:3, and 8:2. The excitation was set at the fluorescence emission maximum of each sample, respectively as pointed in the figure.

**Figure S2.** Plots of Stokes shifts versus $F_{1}(\varepsilon, n)$ (panel a), (v_a_ + v_f_)/2 versus $F_{2}\left( \varepsilon, n \right))$ (panel b) and Stokes shifts versus $E_{T}^{N}$ (panel c) for NTBD in selected solvents: 1 – THF, 2 – ethyl acetate, 3 – chloroform, 4 – acetone, 5 – DMF, 6 – DMSO, 7 – acetonitrile, 8 – propan-2-ol, 9 – butan-1-ol, 10 – ethanol, 11 – methanol. Estimations of dipole moment were performed for two different positions of maximum absorbance, **I** ~ 320 nm and **II** ~ 286 nm.

**Figure S3.** Fluorescence lifetimes (panel a), fractional intensities (panel b) and appropriate standard deviation measured for NTBD at various DMSO : H_2_O volume ratios using 450 nm filter.

**Figure S4.** Panel a: Fluorescence quantum yield for NTBD in various solvents as a function of dielectric constant. Panel b: Fluorescence quantum yield for NTBD in various mixed DMSO : H_2_O solutions as a function of DMSO : H_2_O volume ratio.

**Figure S5.** Fluorescence excitation spectra for NTBD in water solution at varying pH (from 2 to 12).

**Figure S6.** RLS spectra for NTBD in water solution with varying pH (from 2 to 12). The inset presents RLS intensity at 450 nm versus pH.

**Table S1.** Spectroscopic data for NTBD dissolved in different solvents: maximum absorbance and fluorescence. Physical constants of solvents: dielectric constant ε, index of refraction n, functions F_1_(ε,n), F_2_(ε,n), E_T_(30) and $E_{T}^{N}$.

| **Solvent** | **Absorbance**  **(nm)** | **Fluorescence**  **(nm)** | **ε** | **n** | **E_T_(30)** | $\mathbf{E}_{\mathbf{T}}^{\mathbf{N}}$ | **F_1_(ɛ,n)** | **F_2_(ɛ,n)** |
| --- | --- | --- | --- | --- | --- | --- | --- | --- |
| **Methanol** | 321 | 373 | 33 | 1.3265 | 55.5 | 0.765 | 0.856 | 0.502 |
|  | 286 |  |  |  |  |  |  |  |
| **Ethanol** | 322 | 374 | 25.3 | 1.3594 | 51.9 | 0.654 | 0.817 | 0.490 |
|  | 286 |  |  |  |  |  |  |  |
| **Propan-2-ol** | 324 | 373 | 20.18 | 1.3772 | 48.6 | 0.552 | 0.781 | 0.476 |
|  | 286 |  |  |  |  |  |  |  |
| **Butan-1-ol** | 325 | 375 | 17.8 | 1.3993 | 49.7 | 0.586 | 0.753 | 0.467 |
|  | 286 |  |  |  |  |  |  |  |
| **Acetone** | 325 | 365 | 21 | 1.3587 | 42.2 | 0.355 | 0.792 | 0.478 |
| **DMSO** | 320 | 375 | 47.24 | 1.4773 | 45.1 | 0.444 | 0.842 | 0.529 |
|  | 287 |  |  |  |  |  |  |  |
| **DMF** | 320 | 373 | 36.7 | 1.4305 | 43.2 | 0.386 | 0.836 | 0.515 |
|  | 286 |  |  |  |  |  |  |  |
| **Acetonitrile** | 322 | 365 | 36.64 | 1.3416 | 45.6 | 0.460 | 0.862 | 0.508 |
|  | 286 | 365/445 |  |  |  |  |  |  |
| **Cyclohexane** | 330 | 490 | 2.02 | 1.4262 | 30.9 | 0.006 | -0.003 | 0.095 |
|  | 285 |  |  |  |  |  |  |  |
| **Ethyl acetate** | 324 | 365 | 6.02 | 1.3723 | 38.1 | 0.228 | 0.493 | 0.331 |
|  | 288 | 359 |  |  |  |  |  |  |
| **THF** | 325 | 364 | 7.6 | 1.404 | 37.4 | 0.207 | 0.551 | 0.367 |
|  | 290 |  |  |  |  |  |  |  |
| **Toluen** | 326 | 504 | 2.38 | 1.4969 | 33.9 | 0.099 | 0.029 | 0.126 |
|  | 290 | 405 |  |  |  |  |  |  |
| **Chloroform** | 326 | 370/505 | 4.81 | 1.4429 | 39.1 | 0.259 | 0.372 | 0.286 |
|  | 286 | 370/505 |  |  |  |  |  |  |
| ***n*-Hexane** | 327 | 490 | 1.89 | 1.3723 | 30.9 | 0.006 | 0.002 | 0.085 |
|  | 280 | 410/490 |  |  |  |  |  |  |
| ***n*-Heptane** | 326 | 490 | 1.92 | 1.3876 | 31.1 | 0.012 | -0.001 | 0.087 |
|  | 286 | 490 |  |  |  |  |  |  |

**Table S2.** Area under the emission curve I, value of absorbance at the excitation wavelength $\lambda$_ex_ and fluorescence quantum yield $\Phi$_F_ for NTBD in various solvents and mixed DMSO : H_2_O systems.

| **Ratio DMSO:H_2_O** | | **I** | | $\boldsymbol{\lambda}$**_ex_ [nm]** | | $\boldsymbol{\Phi}$**_F_** | |
| --- | --- | --- | --- | --- | --- | --- | --- |
| 1:9 | | 4139.5312 | | 286 | | 0.0573 | |
| 2:8 | | 6265.0884 | | 287 | | 0.0866 | |
| 3:7 | | 9415.9464 | | 287 | | 0.1308 | |
| 4:6 | | 12375.0933 | | 287 | | 0.1722 | |
| 5:5 | | 13836.7440 | | 287 | | 0.1936 | |
| 6:4 | | 12338.0256 | | 287 | | 0.1727 | |
| 7:3 | | 12792.8697 | | 287 | | 0.1790 | |
| 8:2 | | 14915.8511 | | 287 | | 0.2077 | |
| 9:1 | | 18791.6916 | | 287 | | 0.2594 | |
| 1:99 | | 1930.1631 | | 287 | | 0.0265 | |
| 99:1 | | 27310.2024 | | 287 | | 0.3735 | |
| **Solvent** | **I** | | $\boldsymbol{\lambda}$**_ex_ [nm]** | | $\boldsymbol{\Phi}$**_F_** | |  |
| Methanol | 62276.56336 | | 300 | | 0.8223 | |  |
| Ethanol | 53525.33846 | | 302 | | 0.7315 | |  |
| DMF | 31381.14259 | | 300 | | 0.4774 | |  |
| THF | 8864.36259 | | 305 | | 0.1286 | |  |
| Chloroform | 169.8652 | | 307 | | 0.0026 | |  |

**Computational details**

All calculations were performed employing density functional theory (DFT) and its time-dependent variant (TD-DFT) methods with the Gaussian 16 package, version C.01.^^[[1]](#endnote-1)^^ Polarizable continuum model (PCM), with default parameters of the Gaussian/PCM implementation,^^[[2]](#endnote-2)^,^[[3]](#endnote-3)^,^[[4]](#endnote-4)^,^[[5]](#endnote-5)^,^[[6]](#endnote-6)^^ was used to model solvent effects (dimethylsulfoxide = DMSO, ε = 46.826). Symmetry was no explicitly imposed in any computations.

Geometry optimizations of NTBD in its enolic (*cis* and *trans*) and keto forms were carried out using the global hybrid B3LYP^^[[7]](#endnote-7)^,^[[8]](#endnote-8)^,^[[9]](#endnote-9)^^ exchange-correlation functional and split-valence triple-zeta basis set with one set of polarization functions and one set of diffuse functions for all atoms 6-311++G(d,p).^^[[10]](#endnote-10)^,^[[11]](#endnote-11)^^ Dispersion effects were accounted for in these calculations *via* the third-generation Grimme’s set of semiempirical dispersion corrections with the Becke-Johnson damping, D3.^^[[12]](#endnote-12)^,^[[13]](#endnote-13)^^ All geometry optimizations were followed by frequencies calculations at the same level of theory in order to confirm that obtained structures represent energy minima (no imaginary frequencies) and also to evaluate free energy values. Energetic preferences obtained with B3LYP+D3 were further verified by single-point energy calculations employing the dispersion-corrected double-hybrid functional DSDPBEP86^[[14]](#endnote-14)^ (that is expected to provide more accurate energy values)^[[15]](#endnote-15)^ and the 6-311++G(d,p) basis set.

The absorption spectra and emission properties *via* S_1_ excited-state geometry optimizations were computed with B3LYP and the Dunning correlation-consistent polarized valence double-zeta basis set augmented with diffuse functions, aug-cc-pVDZ.^^[[16]](#endnote-16)^,^^[[17]](#endnote-17)^ The presented spectra were simulated based on energies and associated dipole strengths for 100 lowest singlet excited states, as the sums of Gaussian functions centred at the vertical excitation energies and scaled using the calculated oscillator strengths with a broadening parameter of σ = 0.15 eV.^^[[18]](#endnote-18)^^

**Additional computed results**


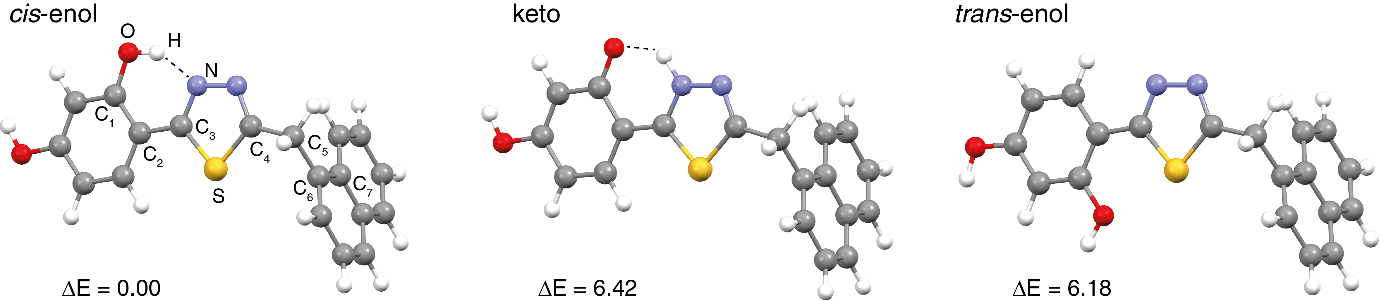


**Figure S7.** DFT-optimized (B3LYP+D3/6-311++G(d,p) with continuum solvent model for DMSO) lowest-energy structures of enolic (*cis* and *trans*) and keto forms of NTBD. Δ*E* values listed are corresponding relative energies computed with respect to the (lowest-energy) *cis*-enol structure, in kcal/mol. See also Table S3.

**Table S3.** Relative energy Δ*E* and relative free energy Δ*G* values computed with respect to the (lowest-energy) *cis*-enol structure along with corresponding selected structural parameters (interatomic distances *d* and dihedral angles ∠) calculated for different lowest-energy structures of NTBD in its enolic (*cis* and *trans*) and keto forms. For structures visualization and atoms labelling, see Figure S7. Based on DFT calculations performed with B3LYP+D3/6‑311++G(d,p)/PCM(DMSO); in parentheses Δ*E* values obtained with DSDPBEP86/6-311++G(d,p)/PCM(DMSO) at B3LYP+D3/6-311++G(d,p)/PCM(DMSO)-optimized structures are listed.

|  | ***cis*-enol** | **keto** | ***trans*-enol** |
| --- | --- | --- | --- |
| **Δ*E* / kcal/mol** | 0.00 (0.00) | 6.42 (9.24) | 6.18 (5.42) |
| **Δ*G* / kcal/mol** | 0.00 | 6.23 | 5.64 |
| ***d*_OH_ / Å** | 0.992 | 1.729 | 0.965 |
| ***d*_NH_ / Å** | 1.726 | 1.038 | – |
| **∠C_1_C_2_C_3_S / deg** | -178.2 | -178.6 | 0.9 |
| **∠SC_4_C_5_C_6_ / deg** | 38.8 | 37.7 | 40.8 |
| **∠C_4_C_5_C_6_C_7_ / deg** | 67.2 | 68.0 | 67.4 |

**
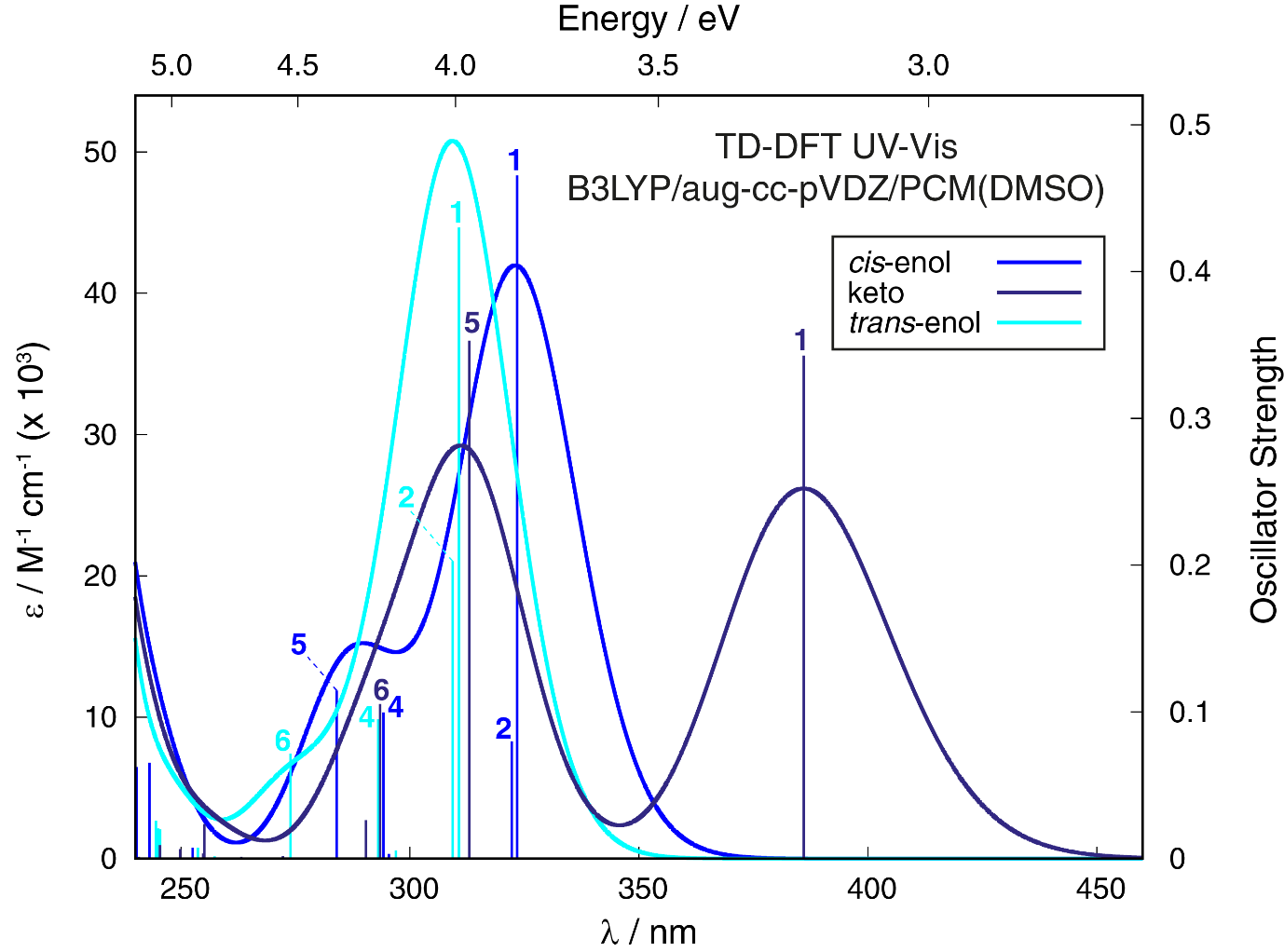
**

**Figure S8.** TD-DFT-simulated UV-Vis spectra for NTBD in its enolic (*cis* and *trans*) and keto forms (for structures visualization, see Figure S7). No spectral shift has been applied. Calculated excitation energies along with corresponding oscillator strengths are indicated as ‘stick’ spectra. Numbered excitations correspond to those analyzed in detail (see Table S4 and Figure S9). Based on calculations performed with B3LYP/aug-cc-pVDZ/PCM(DMSO) at B3LYP+D3/6-311++G(d,p)/PCM(DMSO)-optimized structures.

**Table S4.** Selected excitations and occupied (occ) – unoccupied (unocc) MO-pair contributions (greater than 5%) for NTBD in its enolic (*cis* and *trans*) and keto forms. H and L indicate HOMO and LUMO, respectively. For structures visualization, see Figure S7; for corresponding simulated UV-Vis spectra, see Figure S8; for MOs isosurfaces, see Figure S9. Based on calculations performed with B3LYP/aug-cc-pVDZ/PCM(DMSO) at B3LYP+D3/6‑311++G(d,p)/PCM(DMSO)-optimized structures.

| **Excitation** | ***E* / eV** | **λ / nm** | ***f*** | **occ no.** | **unocc no.** | **%** |
| --- | --- | --- | --- | --- | --- | --- |
| ***cis*-enol** | | | | | | |
| 1 | 3.834 | 323 | 0.466 | 87 (H) | 88 (L) | 85.7 |
|  |  |  |  | 86 | 88 | 9.0 |
| 2 | 3.848 | 322 | 0.080 | 86 | 88 | 89.8 |
|  |  |  |  | 87 | 88 | 8.4 |
| 4 | 4.222 | 294 | 0.100 | 86 | 89 | 91.3 |
| 5 | 4.367 | 284 | 0.115 | 85 | 88 | 83.9 |
|  |  |  |  | 87 | 91 | 7.6 |
| **keto** | | | | | | |
| 1 | 3.212 | 386 | 0.343 | 87 | 88 | 97.4 |
| 5 | 3.962 | 313 | 0.353 | 85 | 88 | 93.4 |
| 6 | 4.225 | 293 | 0.105 | 86 | 89 | 92.5 |
| ***trans*-enol** | | | | | | |
| 1 | 3.991 | 311 | 0.430 | 87 | 88 | 75.6 |
|  |  |  |  | 86 | 88 | 16.9 |
| 2 | 4.008 | 309 | 0.203 | 86 | 88 | 81.1 |
|  |  |  |  | 87 | 88 | 15.3 |
| 4 | 4.231 | 293 | 0.095 | 86 | 89 | 90.6 |
| 6 | 4.527 | 274 | 0.072 | 85 | 88 | 50.6 |
|  |  |  |  | 87 | 91 | 21.5 |
|  |  |  |  | 84 | 88 | 15.2 |


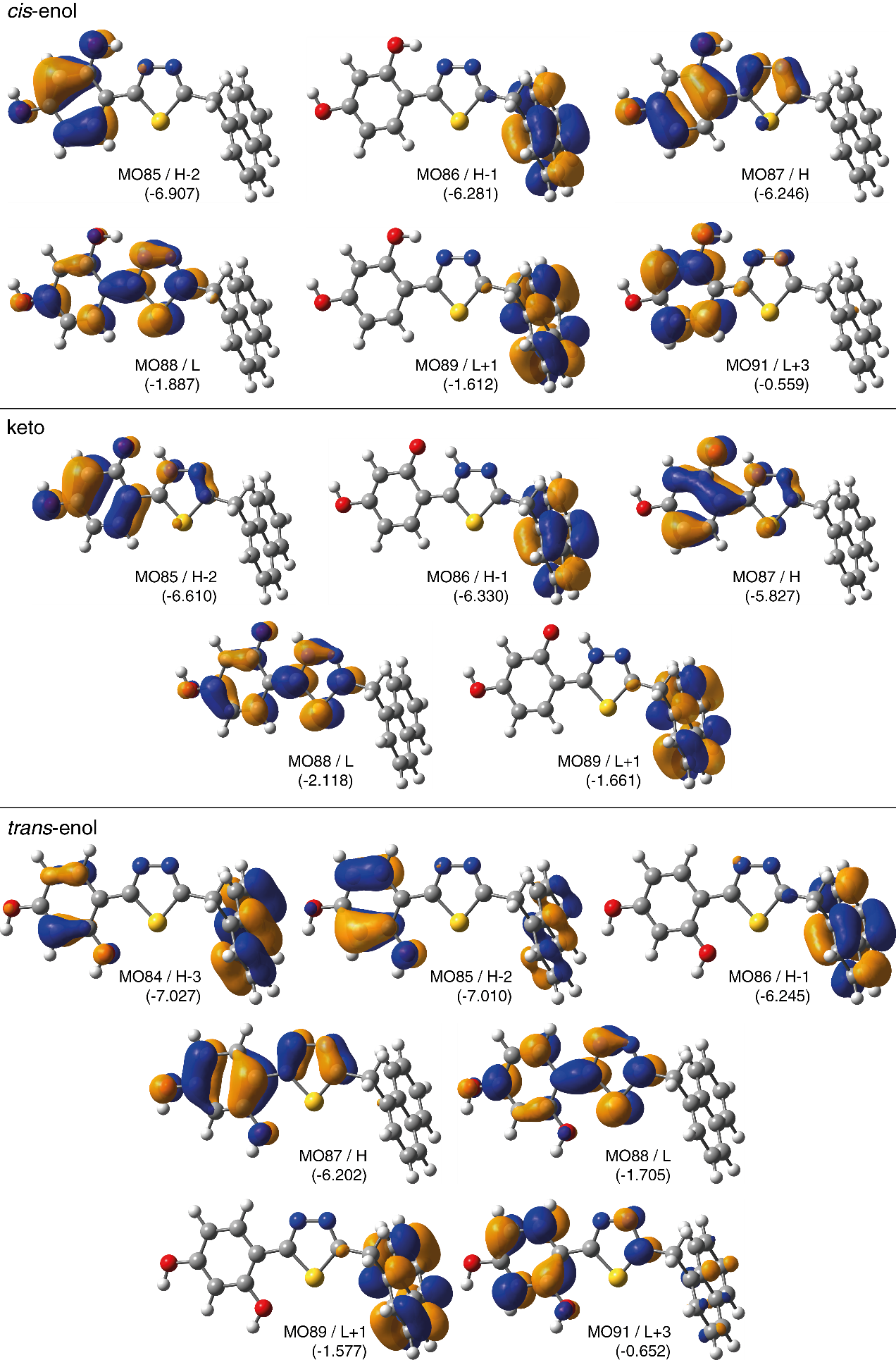


**Figure S9.** Isosurfaces (±0.04 au) of MOs involved in selected electronic transitions of NTBD in its enolic (*cis* and *trans*) and keto forms. H = HOMO, L = LUMO. Values listed in parentheses are corresponding­ orbital energies, in eV. Based on calculations performed with B3LYP/aug-cc-pVDZ/PCM(DMSO) at B3LYP+D3/6-311++G(d,p)/PCM(DMSO)-optimized structures.


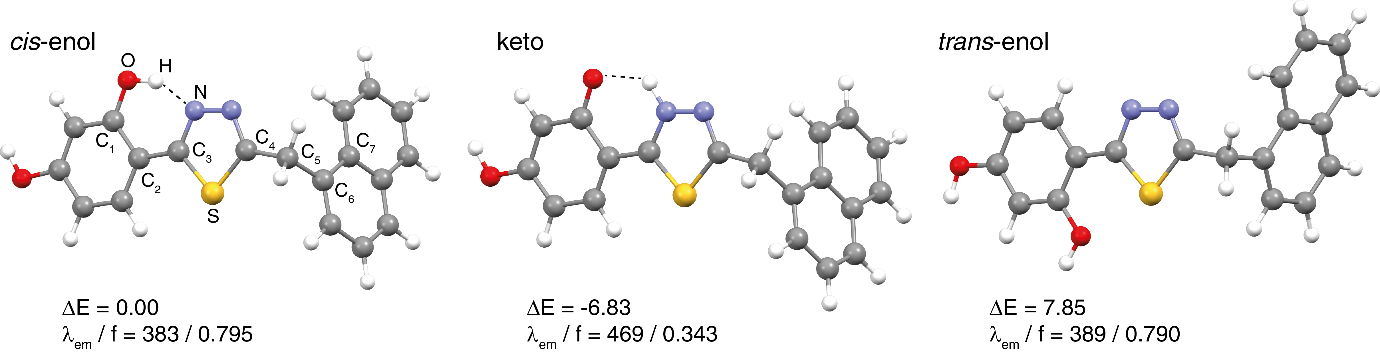


**Figure S10.** TD-DFT-optimized (B3LYP/aug-cc-pVDZ with continuum solvent model for DMSO) lowest-energy singlet excited-state structures of enolic (*cis* and *trans*) and keto forms of NTBD. Values listed correspond to relative energies Δ*E* computed with respect to the *cis*-enol structure (in kcal/mol), fluorescence energies λ_em_ computed as S_1_-S_0_ energy differences at TD-DFT-optimized S_1_ geometries (in nm) and corresponding oscillator strengths *f*. See also Table S5.

**Table S5.** Calculated (TD-DFT B3LYP/aug-cc-pVDZ/PCM(DMSO)) characteristics of S_1_ structures and S_1_→S_0_ fluorescence transitions for NTBD in its enolic (*cis* and *trans*) and keto forms: relative energies Δ*E* computed with respect to the *cis*-enol structure, selected structural parameters (interatomic distances *d* and dihedral angles ∠), fluorescence energies λ_em_ computed as S_1_-S_0_ energy differences at TD-DFT-optimized S_1_ geometries along with corresponding oscillator strengths *f* and percentage contributions of LUMO→HOMO transition to the S_1_→S_0_ emission % L→H. For structures visualization and atoms labelling, see Figure S6; for MOs isosurfaces, see Figure S11.

|  | ***cis*-enol** | **keto** | ***trans*-enol** |
| --- | --- | --- | --- |
| **Δ*E* / kcal/mol** | 0.00 | -6.83 | 7.85 |
| ***d*_OH_ / Å** | 1.010 | 1.954 | 0.968 |
| ***d*_NH_ / Å** | 1.666 | 1.022 | – |
| **∠C_1_C_2_C_3_S / deg** | 179.6 | 178.2 | -0.9 |
| **∠SC_4_C_5_C_6_ / deg** | 66.5 | 52.9 | 88.6 |
| **∠C_4_C_5_C_6_C_7_ / deg** | 77.7 | 73.0 | 92.5 |
| **λ_em_ / nm** | 383 | 469 | 389 |
| ***f*** | 0.795 | 0.343 | 0.790 |
| **% L→H** | 98.1 | 99.4 | 98.7 |


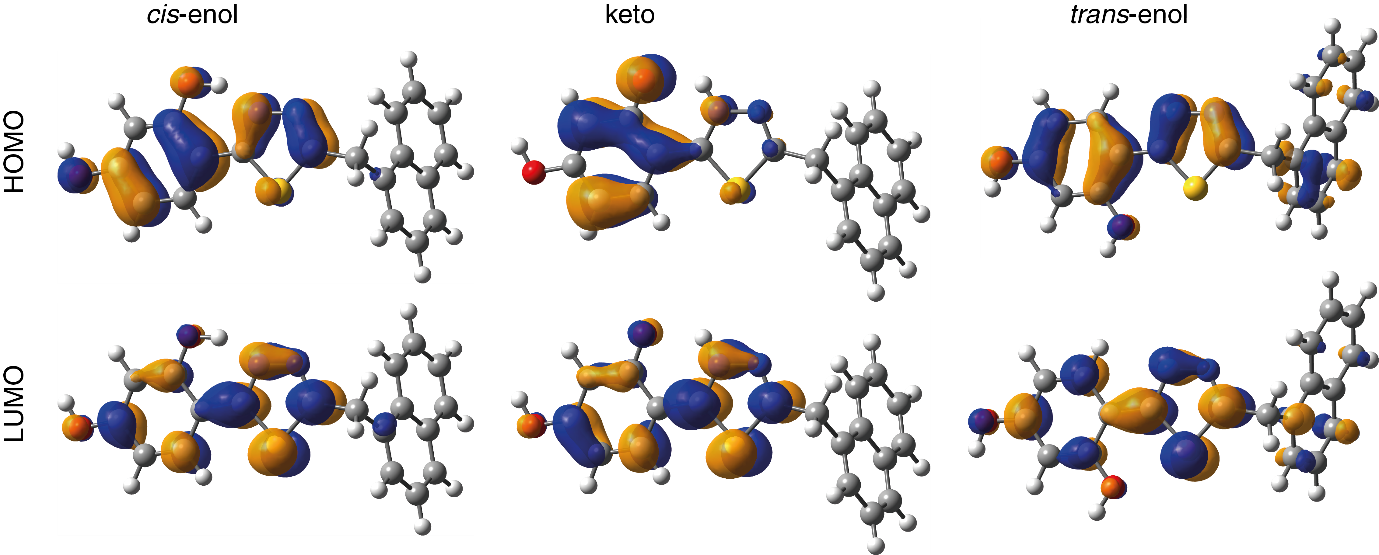


**Figure S11.** Isosurfaces (±0.04 au) of MOs of S_0_ at S_1_ TD-DFT-optimized excited-state geometries predominantly involved in the S_1_→S_0_ transitions for NTBD in its enolic (*cis* and *trans*) and keto forms, obtained based on B3LYP/aug-cc-pVDZ/PCM(DMSO) calculations.

**Cartesian coordinates for optimized structures**

Optimized (B3LYP+D3/6-311++G(d,p) with continuum solvent model for DMSO)) geometries of lowest-energy ground-state structures of NTBD in its enolic (*cis* and *trans*) and keto forms along with the corresponding absolute energies.

The atomic symbol followed by three Cartesian coordinates, in Å.

*cis*-enol

Total energy = -1390.993795 au

C 0.599266 -0.198305 -0.262733

C 0.873465 -1.557426 -0.026320

C 2.163357 -2.044091 0.029487

C 3.233202 -1.155939 -0.155220

C 3.002425 0.193872 -0.394596

C 1.695968 0.682831 -0.450270

H 0.049194 -2.246243 0.116418

H 2.362335 -3.091528 0.212629

O 4.488052 -1.677712 -0.089405

H 3.824109 0.885549 -0.540217

O 1.534428 2.000022 -0.684008

C -0.757546 0.310069 -0.311522

S -2.199595 -0.651539 -0.053245

C -3.115621 0.822238 -0.308765

N -2.374716 1.862566 -0.538263

N -1.039628 1.573491 -0.545461

C -4.614909 0.842372 -0.259770

H 0.562405 2.197611 -0.697494

H 5.147131 -0.985346 -0.222787

C -5.187040 -0.046191 0.823157

H -4.920414 1.883789 -0.133325

H -4.998119 0.513521 -1.229371

C -5.882597 -1.183337 0.476648

C -6.404939 -2.056046 1.458790

C -6.222132 -1.781918 2.790560

C -5.512569 -0.621366 3.197194

C -4.986310 0.265966 2.204282

H -6.029847 -1.417654 -0.571761

H -6.947056 -2.942006 1.150356

H -6.615833 -2.446763 3.551343

C -5.312086 -0.322096 4.569730

C -4.278926 1.419176 2.636876

C -4.101221 1.680092 3.975952

C -4.620839 0.801977 4.953824

H -5.715595 -1.000754 5.313171

H -4.472794 1.019237 6.005069

H -3.869373 2.104296 1.906557

H -3.558388 2.565664 4.285005

keto

Total energy = -1390.983572 au

C -4.621150 0.797855 4.956977

C -5.297505 -0.332018 4.563482

C -5.494533 -0.621910 3.188599

C -4.981139 0.281290 2.203374

C -4.289828 1.440398 2.645478

C -4.114452 1.691603 3.986729

C -5.177563 -0.023728 0.820234

C -5.855819 -1.168038 0.463219

C -6.366373 -2.055475 1.438191

C -6.187586 -1.788833 2.771971

C -4.611912 0.877485 -0.255909

C -3.114557 0.849339 -0.305017

S -2.219484 -0.652818 -0.031323

C -0.742498 0.238826 -0.292000

N -1.057975 1.517949 -0.537713

N -2.368129 1.874020 -0.542336

C 0.585465 -0.234839 -0.250966

C 0.874069 -1.598527 0.005969

C 2.160353 -2.063456 0.054644

C 3.224131 -1.148305 -0.158169

C 2.993920 0.185410 -0.413606

C 1.669193 0.716672 -0.474916

O 4.477963 -1.676558 -0.093234

O 1.450648 1.951874 -0.710821

H 0.056183 -2.292435 0.167041

H 2.384472 -3.103420 0.250134

H 3.819831 0.869913 -0.574867

H 5.135928 -0.986620 -0.245754

H -4.915617 1.918060 -0.120687

H -4.993346 0.556282 -1.228664

H -5.998437 -1.396316 -0.587106

H -6.895585 -2.946595 1.122645

H -6.571711 -2.465391 3.527258

H -5.691308 -1.022533 5.301103

H -4.475235 1.007888 6.009935

H -3.891974 2.139642 1.922061

H -3.584043 2.582005 4.303195

H -0.264195 2.168669 -0.692692

*trans*-enol

Total energy = -1390.983944 au

C 0.000000 0.000000 0.000000

C 0.000000 0.000000 1.407802

C 1.249253 0.000000 2.054851

C 2.438016 -0.000176 1.325665

C 2.398011 0.000241 -0.065391

C 1.168711 -0.000002 -0.735623

C -1.276093 0.000289 2.119679

S -1.466271 0.023668 3.868199

C -3.188069 0.011374 3.589801

N -3.510301 0.002365 2.329401

N -2.428868 -0.008580 1.493379

C -4.185380 0.013479 4.712081

C -3.800106 0.932097 5.851450

C -3.780013 2.350701 5.670498

C -3.375877 3.182725 6.763589

C -3.009300 2.584374 7.997870

C -3.040596 1.220885 8.146978

C -3.436698 0.397692 7.067704

C -4.140225 2.973753 4.445834

C -4.103152 4.342277 4.309472

C -3.703031 5.160986 5.389815

C -3.349273 4.590866 6.589303

O 1.260934 0.000002 3.415266

O 3.526060 0.001130 -0.826280

H 3.388561 -0.000386 1.848667

H 1.150391 0.000404 -1.817636

H -0.954587 0.000405 -0.507958

H -4.283669 -1.005201 5.096660

H -5.153335 0.286169 4.284437

H -2.704487 3.224144 8.818647

H -2.760958 0.765689 9.089694

H -3.450140 -0.678471 7.200111

H -3.042241 5.211492 7.424004

H -3.677431 6.237510 5.268391

H -4.382005 4.797755 3.366445

H -4.445451 2.366456 3.604251

H 2.165414 -0.001254 3.751658

H 4.311781 0.002337 -0.265941

**References**

1. Gaussian 16, Revision C.01, M. J. Frisch, G. W. Trucks, H. B. Schlegel, G. E. Scuseria, M. A. Robb, J. R. Cheeseman, G. Scalmani, V. Barone, G. A. Petersson, H. Nakatsuji, X. Li, M. Caricato, A. V. Marenich, J. Bloino, B. G. Janesko, R. Gomperts, B. Mennucci, H. P. Hratchian, J. V. Ortiz, A. F. Izmaylov, J. L. Sonnenberg, D. Williams-Young, F. Ding, F. Lipparini, F. Egidi, J. Goings, B. Peng, A. Petrone, T. Henderson, D. Ranasinghe, V. G. Zakrzewski, J. Gao, N. Rega, G. Zheng, W. Liang, M. Hada, M. Ehara, K. Toyota, R. Fukuda, J. Hasegawa, M. Ishida, T. Nakajima, Y. Honda, O. Kitao, H. Nakai, T. Vreven, K. Throssell, J. A. Montgomery, Jr., J. E. Peralta, F. Ogliaro, M. J. Bearpark, J. J. Heyd, E. N. Brothers, K. N. Kudin, V. N. Staroverov, T. A. Keith, R. Kobayashi, J. Normand, K. Raghavachari, A. P. Rendell, J. C. Burant, S. S. Iyengar, J. Tomasi, M. Cossi, J. M. Millam, M. Klene, C. Adamo, R. Cammi, J. W. Ochterski, R. L. Martin, K. Morokuma, O. Farkas, J. B. Foresman, and D. J. Fox, Gaussian, Inc., Wallingford CT, 2016. [↑](#endnote-ref-1)
2. J. Tomasi, B. Mennucci, R. Cammi, *Chem. Rev.* **2005**, *105*, 2999-3094. [↑](#endnote-ref-2)
3. M. Cossi, V. Barone, R. Cammi, J. Tomasi, *Chem. Phys. Lett.* **1996**, *255*, 327-335. [↑](#endnote-ref-3)
4. G. Scalmani, M. J. Frisch, *J. Chem. Phys.* **2010**, *132*, 114110. [↑](#endnote-ref-4)
5. M. Cossi, V. Barone, *J. Chem. Phys.* **2001**, *115*, 4708-4717. [↑](#endnote-ref-5)
6. G. Scalmani, M. J. Frisch, B. Mennucci, J. Tomasi, R. Cammi, V. Barone, *J. Chem. Phys.* **2006**, *124*, 094107. [↑](#endnote-ref-6)
7. A. D. Becke, *J. Chem. Phys.* **1993**, *98*, 5648-5652. [↑](#endnote-ref-7)
8. C. Lee, W. Yang, R. G. Parr, *Phys. Rev. B*, **1988**, *37*, 785-789. [↑](#endnote-ref-8)
9. P. J. Stephens, F. J. Devlin, C. F. Chabalowski, M. J. Frisch, *J. Phys. Chem.* **1994**, *98*, 11623-11627. [↑](#endnote-ref-9)
10. R. Krishnan, J. S. Binkley, R. Seeger, J. A. Pople, *J. Chem. Phys.* **1980**, *72*, 650-654. [↑](#endnote-ref-10)
11. T. Clark, J. Chandrasekhar, G. W. Spitznagel, P. Von Ragué Schleyer, *J. Comput. Chem.* **1983**, *4*, 294-301. [↑](#endnote-ref-11)
12. S. Grimme, J. Antony, S. Ehrlich, H. Krieg, *J. Chem. Phys.* **2010**, *132*, 154104. [↑](#endnote-ref-12)
13. S. Grimme, S. Ehrlich, L. Goerigk, *J. Comput. Chem.* **2011**, *32*, 1456-1465. [↑](#endnote-ref-13)
14. S. Kozuch, J. M. L. Martin, *Phys. Chem. Chem. Phys.* **2011**, *13*, 20104-20107. [↑](#endnote-ref-14)
15. L. Goerigk, N. Mehta, *Aust. J. Chem.* **2019**, *72*, 563-573. [↑](#endnote-ref-15)
16. T. H. Dunning Jr., *J.* *Chem.* *Phys.* **1989**, *90*, 1007-1023. [↑](#endnote-ref-16)
17. R. A. Kendall, T. H. Dunning Jr., R. J. Harrison, *J. Chem. Phys.* **1992**, *96*, 6796-6806. [↑](#endnote-ref-17)
18. J. Autschbach, T. Ziegler, S. J. A. van Gisbergen, E. J. Baerends, *J. Chem. Phys.* **2002**, *116*, 6930-6940. [↑](#endnote-ref-18)
